# Adaptor protein 3BP2 regulates gene expression in addition to the ubiquitination and proteolytic activity of MALT1 in dectin-1–stimulated cells

**DOI:** 10.1016/j.jbc.2024.107980

**Published:** 2024-11-13

**Authors:** Ayumi Tsubokawa, Kazuyasu Chihara, Yuri Chihara, Kenji Takeuchi, Shigeharu Fujieda, Kiyonao Sada

**Affiliations:** 1 Department of Genome Science and Microbiology, Faculty of Medical Sciences, University of Fukui, Eiheiji, Fukui, Japan; 2 Department of Otorhinolaryngology Head & Neck Surgery, Faculty of Medical Sciences, University of Fukui, Eiheiji, Fukui, Japan; 3 Life Science Innovation Center, University of Fukui, Fukui, Fukui, Japan

**Keywords:** NF-kappa B, ubiquitination, dendritic cells, signal transduction, dectin-1, spleen tyrosine kinase, CARD9, BCL10, MALT1, TRAF6

## Abstract

Dectin-1, a C-type lectin, plays important roles in the induction of antifungal immunity. Caspase recruitment domain-containing protein 9 (CARD9) is essential for the dectin-1–induced production of cytokines through the activation of NF-κB. However, the molecular mechanisms underlying the dectin-1–mediated activation of CARD9 have not been fully elucidated. Recently, we reported that the adaptor protein SH3 domain-binding protein 2 (3BP2) is required for the dectin-1–induced production of cytokines and activation of NF-κB, although the relationship between 3BP2 and CARD9 in dectin-1–mediated signaling remains unclear. Here, we report that 3BP2 is required for dectin-1–induced expression of several genes that may contribute to antifungal immunity in bone marrow–derived dendritic cells (BMDCs). The results of reporter assays using HEK-293T cells indicate that 3BP2 induces CARD9-mediated activation of NF-κB through B-cell leukemia/lymphoma 10, mucosa-associated lymphoid tissue lymphoma translocation protein 1 (MALT1), and TNF receptor-associated factor 6-dependent mechanisms. In addition, we show that 3BP2 induces CARD9-mediated ubiquitination of cellular proteins and that MALT1 cleaves 3BP2 in a CARD9-dependent manner. Furthermore, we show that 3BP2 is required for the ubiquitination, in addition to the activation, of MALT1, which leads to MALT1-depenedent cleavage of 3BP2 in dectin-1–stimulated BMDCs. Finally, we identified hematopoietic cell-specific Lyn substrate 1 as a target of 3BP2, which is essential for dectin-1–induced expression of interleukin 10 in BMDCs. These results indicate that 3BP2 regulates gene expression and functions of MALT1 in dectin-1–stimulated cells and that 3BP2 plays an important role in the dectin-1–mediated antifungal immunity.

Dectin-1 is a C-type lectin receptor primarily expressed on myeloid cells, such as macrophages and dendritic cells, which acts as a pattern recognition receptor in pathogenic fungi (1). It is a type II membrane protein containing a carbohydrate recognition domain at its extracellular C-terminus and a hem-immunoreceptor tyrosine-based activation motif (hemITAM) at its intracellular N-terminus (2). Dectin-1 recognizes β-glucans, such as curdlan, a component of the fungal cell walls (3) to activate spleen tyrosine kinase (Syk) through tyrosine phosphorylation of hemITAM upon ligand binding (4). Activation of Syk through dectin-1 leads to the caspase recruitment domain-containing protein 9 (CARD9)-mediated activation of NF-κB, which is essential for the induction of antifungal immunity (5).

Using gene KO mice, protein kinase C δ (PKCδ) and Vav family guanine nucleotide exchange factors have been shown to be involved in the Syk-dependent regulation of CARD9 in bone marrow–derived dendritic cells (BMDCs) (6, 7). Vav family proteins are involved in the activation of IκB kinase (IKK) α and β collaboratively with the downstream signaling of PKCδ in dectin-1–stimulated BMDCs (7). In addition, the E3 ubiquitin ligase tripartite motif-containing 62 (TRIM62) plays an important role in regulating CARD9 through the ubiquitination of Lys125 in CARD9 (8). However, the molecular mechanisms by which PKCδ, Vav family proteins, and TRIM62 co-operatively regulate the functions of CARD9 have remained elusive.

CARD9 is known to be essential for immunity in mice infected with *Candida albicans* (5, 9) and for the production of cytokines, such as tumor necrosis factor (TNF)-α, interleukin (IL)-2, IL-6, IL-10, and IL-23, in BMDCs stimulated by dectin-1 (10). CARD9 is believed to form molecular complexes together with an adaptor protein known as B-cell leukemia/lymphoma 10 (BCL10) and a caspase-like protease known as mucosa-associated lymphoid tissue lymphoma translocation protein 1 (MALT1) downstream of Syk (9, 11, 12). The formation of molecular complexes is thought to be important for the function of MALT1 as a scaffold protein to interact with a ubiquitin ligase, TNF receptor-associated factor 6 (TRAF6), which induces the ubiquitination of MALT1 and IKK γ for the activation of NF-κB through IKK α and β (13, 14, 15). The protease activity of MALT1 is also believed to be important for the optimal activation of NF-κB (16).

SH3 domain-binding protein 2 (3BP2) is an adaptor protein known as SH3BP2, which contains an N-terminal pleckstrin homology (PH) domain and a C-terminal Src homology (SH) 2 domain. 3BP2 was originally identified as a c-Abl-SH3–binding protein (17). Subsequently, it was demonstrated that 3BP2 interacts with Syk to activate the nuclear factor of activated T cells in T cell lines (18). In addition, 3BP2 is tyrosine-phosphorylated in response to the engagement of B-cell receptors and forms molecular complexes with Syk and Vav (19, 20). Recently, we found that 3BP2 is important for Syk-dependent phagocytosis and chemokine expression induced by the Fc receptor for IgG in a monocytic cell line (21). Using 3BP2-KO mice, 3BP2 was demonstrated to be involved in the phagocytosis of *Escherichia coli* by macrophages (22). In addition, 3BP2 plays important roles in the B-cell receptor–stimulated growth of B cells (23, 24) and bone homeostasis mediated by osteoclasts and osteoblasts (25). In humans, gain-of-function mutations in 3BP2 cause cherubism, an inherited bone disease (26). In a mouse model of cherubism, a cherubism mutation led to the accumulation of 3BP2 and robust activation of Src, Syk, and Vav in osteoclasts (27). To further investigate the physiological roles of 3BP2, we recently generated 3BP2^DL/DL^ mice expressing a 3BP2 mutant (amino acids residues 1–181 with eight extra amino acids) that contains the N-terminal PH domain but no other functional domains because of a frameshift mutation introduced using the CRISPR/Cas9 system. Using 3BP2^DL/DL^ mice, we demonstrated that 3BP2 is essential for the expression of cytokine genes and for the activation of NF-κB in dectin-1–stimulated BMDCs (28). However, the molecular mechanisms underlying the dectin-1–induced gene expression regulated by 3BP2 through CARD9 remain unclear.

In this study, microarray analysis revealed that 3BP2 plays an important role in the dectin-1–induced expression of a variety of genes that might contribute to antifungal immunity. To analyze the molecular mechanism, we performed reporter assays using HEK-293T cells and found that BCL10, MALT1, and TRAF6 are essential for 3BP2-mediated activation of NF-κB *via* CARD9. In addition, we show that 3BP2 induces TRAF6-dependent ubiquitination of cellular proteins in a CARD9-dependent manner, which is likely to couple with the activation of NF-κB. Furthermore, we show that 3BP2 is required for the ubiquitination, in addition to the activation, of MALT1, which induces MALT1-dependent proteolytic cleavage of 3BP2 in dectin-1–stimulated BMDCs. Finally, we identified hematopoietic cell-specific Lyn substrate 1 (HS1), as a target of 3BP2, which is necessary for IL-10 gene expression in dectin-1–stimulated BMDCs.

## Results

### 3BP2 is required for gene expression induced by dectin-1

**Figure 1:**
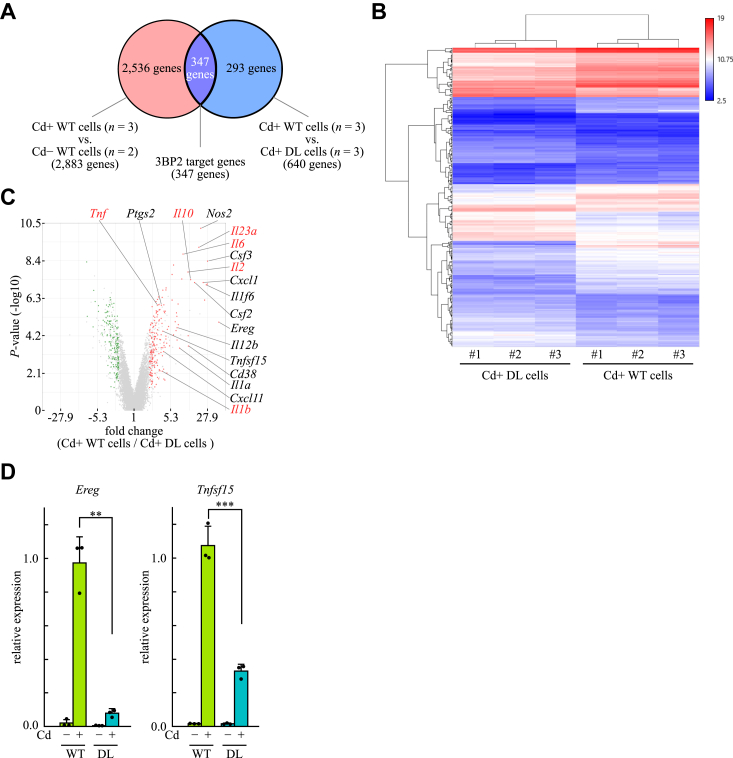
**3BP2 is required for dectin-1–induced gene expression in BMDCs.***A*, Venn diagram showing the definition of 3BP2 target genes. Differentially expressed genes were identified by comparing data collected using BMDCs obtained from WT mice (WT cells) or 3BP2^DL/DL^ mice (DL cells) without (Cd−) or with (Cd+) stimulation with curdlan. *n* represents the number of biological replicates for normalization. *B*, hierarchical clustering of the 3BP2 target genes. Expression levels are colored *red* for high intensity and *blue* for low intensity. Sample names and types of cells are indicated at the *bottom*. *C*, a volcano plot showing differential expression of the 3BP2 target genes in two different genotypes of cells stimulated with curdlan. Gene symbols in *red* were identified as the 3BP2 target genes in our previous study. *D*, after WT or DL cells were stimulated without (Cd−) or with (Cd+) curdlan for 4 h, total RNA was extracted and the mRNA expression of indicated genes was analyzed using qPCR. Closed circles correspond to an individual biological replicate of three independent experiments. Data are presented as the mean ± SD. Statistical significance was analyzed using Welch’s *t* test (*Ereg*) or Student’s *t* test (*Tnfsf15*). ∗∗∗*p* < 0.001 or ∗∗*p* < 0.01 was considered statistically significant.

In a previous study, we reported that 3BP2 plays an important role in dectin-1–induced production of cytokines in BMDCs (28). To further investigate the role of 3BP2 in dectin-1–mediated signaling, we analyzed gene expression in BMDCs obtained from WT (WT cells) and 3BP2^DL/DL^ (DL cells) mice using DNA microarrays. A total of 2883 genes were identified as differentially expressed genes by comparing curdlan-stimulated WT cells with nonstimulated WT cells (red circle, Fig. 1*A* and Table S1). Next, we compared gene expression in curdlan-stimulated WT cells with that in curdlan-stimulated DL cells. A total of 640 genes were identified as differentially expressed genes (blue circle). Finally, 347 genes were identified to be common in both the groups (purple area, Table S2). Because the expression of these genes is thought to be regulated by both dectin-1 and 3BP2, we defined them as 3BP2 target genes. As shown in Figure 1*B*, hierarchal clustering revealed that the expression pattern of the 3BP2 target genes was similar between cell replicates but different between cell genotypes. We further analyzed the expression profile of the 3BP2 target genes using a volcano plot (Fig. 1*C*) and confirmed that 3BP2 is required for dectin-1–induced expression of *Tnf*, *Il1b*, *Il2*, *IL6*, *Il10*, and *Il23a*, as we reported previously (28). In addition, we found that 3BP2 is also required for dectin-1–induced expression of *Cd38* (29), *Csf2* (30), *Csf3* (31), *Cxcl1* (32), *Cxcl11* (33), *Il1a* (32), *Il1f6* (34), *Il12b* (35), *Nos2* (33), *Ptgs2* (36), *and Tnfsf15* (37). Furthermore, we found that the expression of *Ereg* is induced by stimulation with curdlan, which was dependent on 3BP2. Because *Ereg* (38) and *Tnfsf15* (39), as well as *Card9* (40, 41) are involved in the pathogenesis of dextran sulfate sodium (DSS)-induced colitis in mice, it appeared important to verify the expression of these genes using quantitative PCR (qPCR). As shown in Figure 1*D*, the expression of these genes was upregulated in response to stimulation with curdlan, whereas it was dramatically reduced in DL cells compared with that in WT cells. Taken together, these results indicated that 3BP2 is required for gene expression induced by dectin-1. In addition, these results suggested that 3BP2 plays an important role in dectin-1–mediated signaling, which leads to the activation of NF-κB *via* CARD9.

### BCL10, MALT1, and TRAF6, in addition to CARD9, are essential for the 3BP2-mediated activation of NF-κB in HEK-293T cells

**Figure 2:**
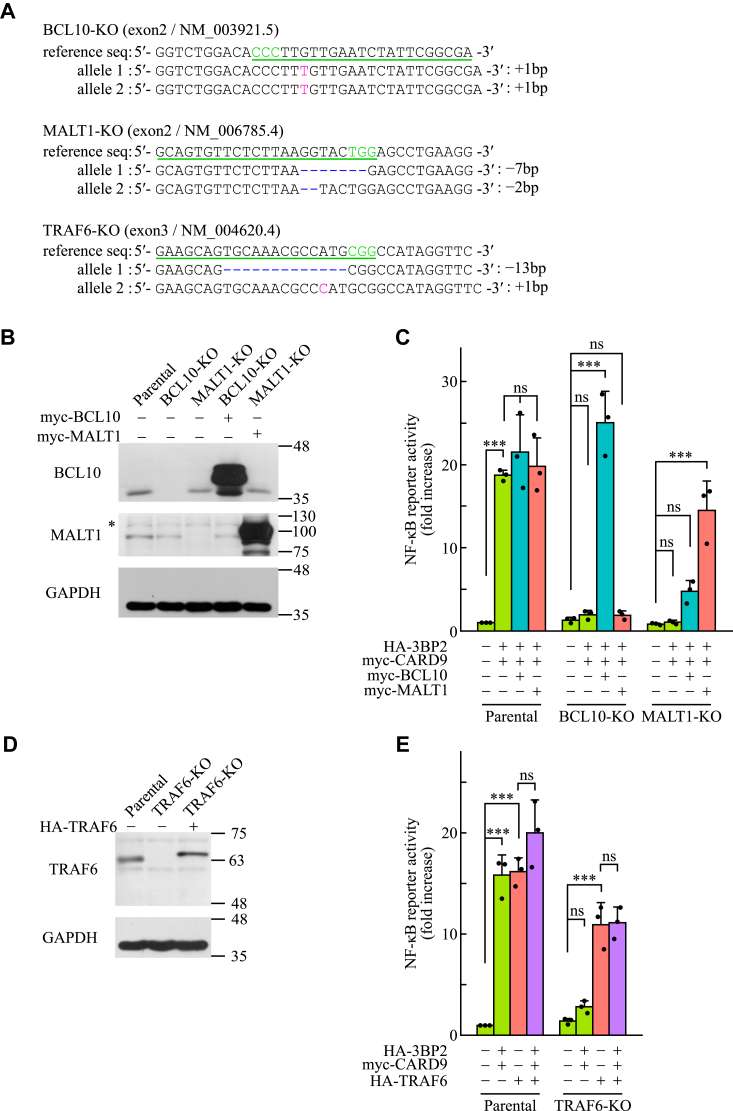
**BCL10, MALT1, and TRAF6, in addition to CARD9, are required for 3BP2-mediated activation of NF-κB in HEK-293T cells.***A*, genomic sequence of BCL10-knockout (KO), MALT1-KO, or TRAF6-KO HEK-293T cells generated using the CRISPR/Cas9 system. Targeted exon and GenBank accession numbers of the indicated genes are shown in parentheses. The sequences of sgRNAs are underlined. Characters in *green* represent the protospacer adjacent motif and those in *red* represent the newly inserted nucleotides. Deleted nucleotides are shown using hyphens in *blue*. *B* and *D*, HEK-293T cells (Parental) or KO cell lines were transfected without (−) or with (+) plasmids encoding the indicated proteins, and the cell lysates were subjected to immunoblotting analysis. Data are representative of an individual biological replicate of three independent experiments. ∗ represents the position of a protein nonspecifically recognized by the primary antibody. *C* and *E*, HEK-293T cells (Parental) or KO cell lines were transfected with luciferase reporter plasmids without (−) or with (+) plasmids encoding the indicated proteins, and the cell lysates were subjected to a luciferase assay. Normalized luciferase activities are expressed as fold increases compared to that observed in cells expressing GFP. Closed circles correspond to an individual biological replicate of three independent experiments. Data are presented as the mean ± SD. Statistical significance was analyzed using two-way ANOVA followed by the Tukey–Kramer test. ∗∗∗*p* < 0.001 was considered statistically significant. ns = not significant.

Our previous study revealed that 3BP2 induces CARD9-mediated NF-κB activation in HEK-293T cells (28). To further explore the relationship between 3BP2 and CARD9-mediated signaling, we generated BCL10-KO, MALT1-KO, and TRAF6-KO cells using the CRISPR/Cas9 system. Genomic DNA of these cells was extracted, and the frameshift mutations induced by the CRISPR/Cas9 system were verified using DNA sequencing (Fig. 2*A*). We also analyzed the expression of BCL10 and MALT1 proteins in parental, BCL10-KO, and MALT1-KO cells using immunoblotting analysis. As shown in Figure 2*B*, the endogenous expression of BCL10 and MALT1 was abrogated in BCL10-KO or MALT1-KO cells. Luciferase assay was performed on these cells. Although overexpression of 3BP2 alongside CARD9 significantly increased NF-κB reporter activity in parental HEK-293T cells, this NF-κB reporter activity in BCL10-KO or MALT1-KO cells overexpressing 3BP2 with CARD9 was severely reduced to almost the basal level (Fig. 2*C*). In addition, the exogenous expression of BCL10 or MALT1 significantly rescued the activation of NF-κB in BCL10-KO and MALT1-KO cells. These results indicate that BCL10, MALT1, and CARD9 are essential for the 3BP2-mediated activation of NF-κB in HEK-293T cells.

As immunoblotting analysis showed that the endogenous expression of TRAF6 was abrogated in TRAF6-KO cells (Fig. 2*D*), we performed luciferase assays using these cells. As shown in Figure 2*E*, the NF-κB reporter activity in TRAF6-KO cells overexpressing 3BP2 with CARD9 was dramatically reduced to almost the basal level. On the contrary, as previously reported (42), we found that overexpression of TRAF6 induced the NF-κB reporter activity in both parental and TRAF6-KO cells. However, coexpression of TRAF6 with 3BP2 and CARD9 could not further enhance the NF-κB reporter activity in both the cell types. These results suggested that TRAF6 activates NF-κB through a common signaling pathway regulated by 3BP2 *via* CARD9.

### 3BP2 accelerates TRAF6-dependent ubiquitination of cellular proteins and is subjected to MALT1-dependent cleavage in HEK-293T cells overexpressing CARD9

**Figure 3:**
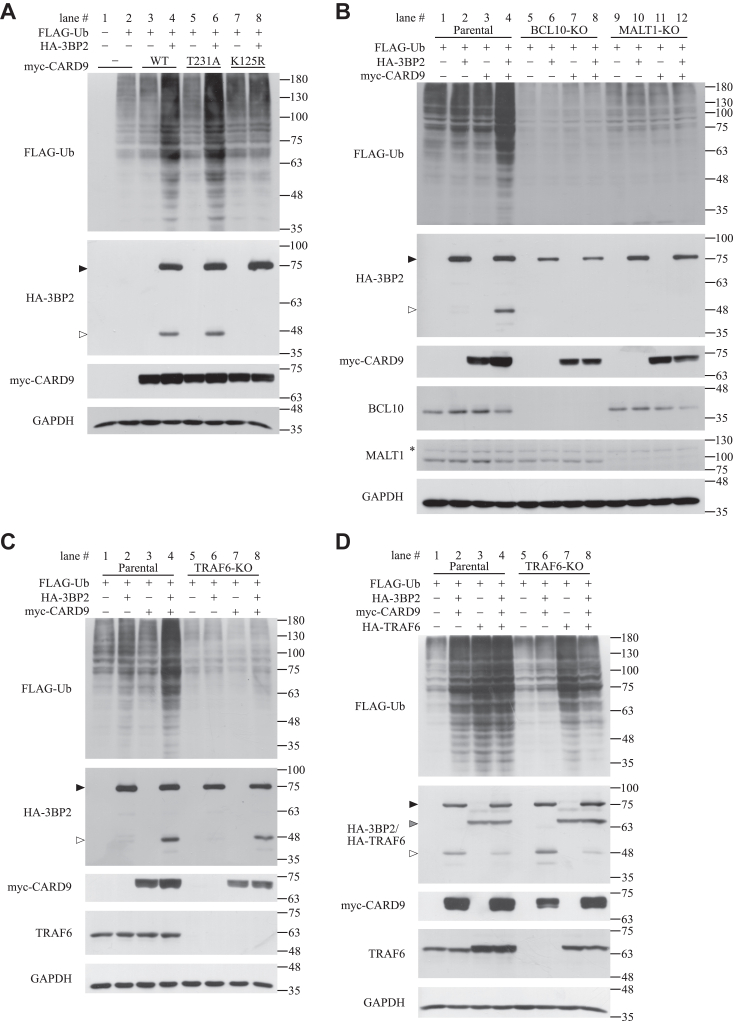
**3BP2 induces TRAF6-dependent ubiquitination of cellular proteins and is subjected to MALT1-dependent cleavage in HEK-293T cells overexpressing CARD9.***A*–*D*, HEK-293T cells (Parental) or KO cell lines were transfected without (−) or with (+) plasmids encoding the indicated proteins, and the cell lysates were subjected to immunoblotting analysis. The positions of uncleaved HA-3BP2, HA-TRAF6, and cleaved HA-3BP2 are indicated by *closed*, *gray*, and *open* arrowheads, respectively. ∗ represents the position of a protein nonspecifically recognized by the primary antibody. Data are representative of an individual biological replicate of three independent experiments.

Various signaling molecules such as MALT1 are believed to be modified by ubiquitin under the control of CARD9 (9, 13, 15, 43). To examine whether 3BP2 is involved in protein ubiquitination, FLAG-tagged ubiquitin, 3BP2, and mutant forms of CARD9 were overexpressed in HEK-293T cells, and cell lysates were subjected to immunoblotting analysis. Previous studies have demonstrated that PKCδ phosphorylates Thr231 on CARD9, and a T231A mutation in CARD9 abrogates IL-10 expression in dectin-1–stimulated BMDCs (6). Similarly, the K125R mutation in CARD9 has been demonstrated to dramatically reduce NF-κB activation in dectin-1–stimulated THP-1 cells (8). As shown in Figure 3*A*, the overexpression of 3BP2 with CARD9 (lane 4), but not CARD9 alone (lane 3), accelerated the modification of cellular proteins with FLAG-tagged ubiquitin. In addition, the 3BP2-mediated ubiquitination of cellular proteins was not influenced by the T231A mutation in CARD9 (lane 6), whereas it was abrogated by the K125R mutation (lane 8). These results indicated that phosphorylation of Thr231 by PKCδ is not essential for the 3BP2-mediated ubiquitination of cellular proteins. Interestingly, we detected additional bands that were recognized by anti-HA antibody in the lysates of cells overexpressing 3BP2 with CARD9 (lane 4) or the T231A mutant (lane 6), but not the K125R mutant (lane 8).

Next, we examined whether BCL10 or MALT1, in addition to CARD9, is required for the 3BP2-mediated ubiquitination of cellular proteins. 3BP2-mediated ubiquitination of cellular proteins was almost abrogated in BCL10-KO or MALT1-KO cells (Fig. 3*B*, lane 4 vs. 8 or 12). The importance of BCL10 and MALT1 was validated by data showing that exogenous expression of BCL10 or MALT1 in these KO cells rescued the 3BP2-mediated ubiquitination of cellular proteins (Fig.S1, lane 6 vs. 7, and lane 10 vs. 12). Importantly, an additional band recognized by anti-HA antibody was not observed in KO cells overexpressing 3BP2 with CARD9 (Fig. 3*B*, lanes 8 and 12). Because MALT1 is believed to act as a protease that is activated through the formation of complexes with BCL10 and CARD9 (9, 14, 16, 44), we hypothesized that the additional band was part of the HA-tagged 3BP2 cleaved by MALT1. Taken together, these results suggest that BCL10 and MALT1, in addition to CARD9, play important roles in 3BP2-mediated ubiquitination of cellular proteins and cleavage of 3BP2.

Using TRAF6-KO cells, we examined whether TRAF6 is involved in the 3BP2-mediated ubiquitination of cellular proteins. As shown in Figure 3*C*, 3BP2-mediated ubiquitination of cellular proteins was almost completely abrogated in TRAF6-KO cells (lane 8), as observed in BCL10-KO or MALT1-KO cells (Fig. 3*B*, lanes 8 and 12). Importantly, the cleavage of HA-tagged 3BP2 was not influenced by the absence of endogenous TRAF6 (lanes 4 and 8). Next, we analyzed the effects of TRAF6 overexpression on the ubiquitination of cellular proteins and cleavage of 3BP2. As shown in Figure 3*D*, overexpression of TRAF6 strongly induced the ubiquitination of cellular proteins in both parental and TRAF6-KO cells (lanes 3 and 7), whereas this was not further accelerated by coexpression of 3BP2 with CARD9 (lanes 4 and 8). Notably, the amount of cleaved 3BP2 was slightly decreased by the overexpression of TRAF6 in these cells (lane 2 vs. 4, and lane 6 vs. 8). Taken together, these results suggest that TRAF6 is required and sufficient for 3BP2-mediated ubiquitination of cellular proteins but is not essential for MALT1-dependent cleavage of 3BP2 in HEK-293T cells.

### Cleavage of 3BP2 by MALT1 is accelerated by overexpression of FLAG-tagged ubiquitin in a CARD9-dependent manner

**Figure 4:**
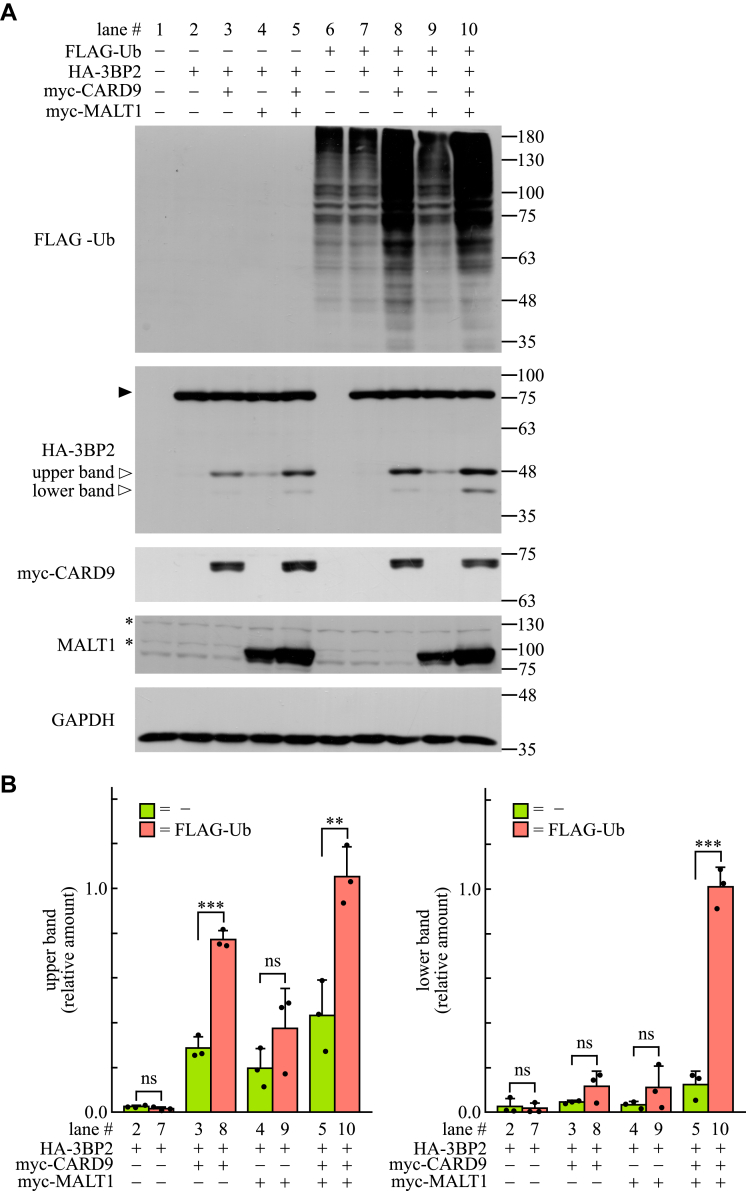
**Overexpression of FLAG-tagged ubiquitin together with CARD9 accelerates the MALT1-dependent cleavage of 3BP2 in HEK-293T cells.***A*, HEK-293T cells were transfected without (−) or with (+) plasmids encoding the indicated proteins, and the cell lysates were subjected to immunoblotting analysis. The positions of uncleaved or cleaved HA-3BP2 are indicated by *closed* or *open* arrowheads (*upper* and *lower* bands), respectively. Data are representative of an individual biological replicate of three independent experiments. ∗ represents the position of a protein nonspecifically recognized by the primary antibody. *B*, relative amount of cleaved 3BP2 (relative to GAPDH) was quantified using the immunoblots shown in panel *A*. Closed circles correspond to an individual biological replicate of three independent experiments. Data are presented as the mean ± SD. Statistical significance was analyzed using Welch’s *t* test. ∗∗∗*p* < 0.001 or ∗∗*p* < 0.01 was considered statistically significant. ns = not significant.

Ubiquitination of MALT1 plays important roles in the CARD9-mediated activation of NF-κB as well as in the intrinsic protease activity (14, 15, 45). To investigate the relationship between the ubiquitination of cellular proteins and MALT1-dependent cleavage of 3BP2, HEK-293T cells were transfected with plasmids encoding HA-tagged 3BP2, FLAG-tagged ubiquitin, CARD9, and MALT1. Cell lysates were subjected to immunoblotting analysis. As shown in Figure 3, cleavage of HA-tagged 3BP2 was observed in cells overexpressing CARD9 (Fig. 4*A*, lane 3, upper band). In addition, we found that the overexpression of CARD9 with FLAG-tagged ubiquitin significantly increased the amount of cleaved fragment of HA-tagged 3BP2 (Fig. 4, *A* and *B*, lane 3 vs. 8, upper band). The effect of FLAG-tagged ubiquitin overexpression was also observed in cells overexpressing CARD9 and MALT1 (lane 5 vs. 10), but not in the absence of CARD9 (lane 4 vs. 9).

Importantly, we detected another cleaved fragment of HA-tagged 3BP2 in the lysates obtained from cells overexpressing CARD9 and MALT1 (Fig. 4*A*, lanes 5 and 10, lower band). Quantitative analyses revealed that the intensity of the lower band was dramatically increased by the overexpression of FLAG-tagged ubiquitin (Fig. 4*B*, lane 5 vs. 10). These results indicate that overexpression of FLAG-tagged ubiquitin enhances the protease activity of MALT1 and accelerates the cleavage of 3BP2 in a CARD9-dependent manner.

### 3BP2 is essential for the ubiquitination of MALT1 in dectin-1–stimulated BMDCs

**Figure 5:**
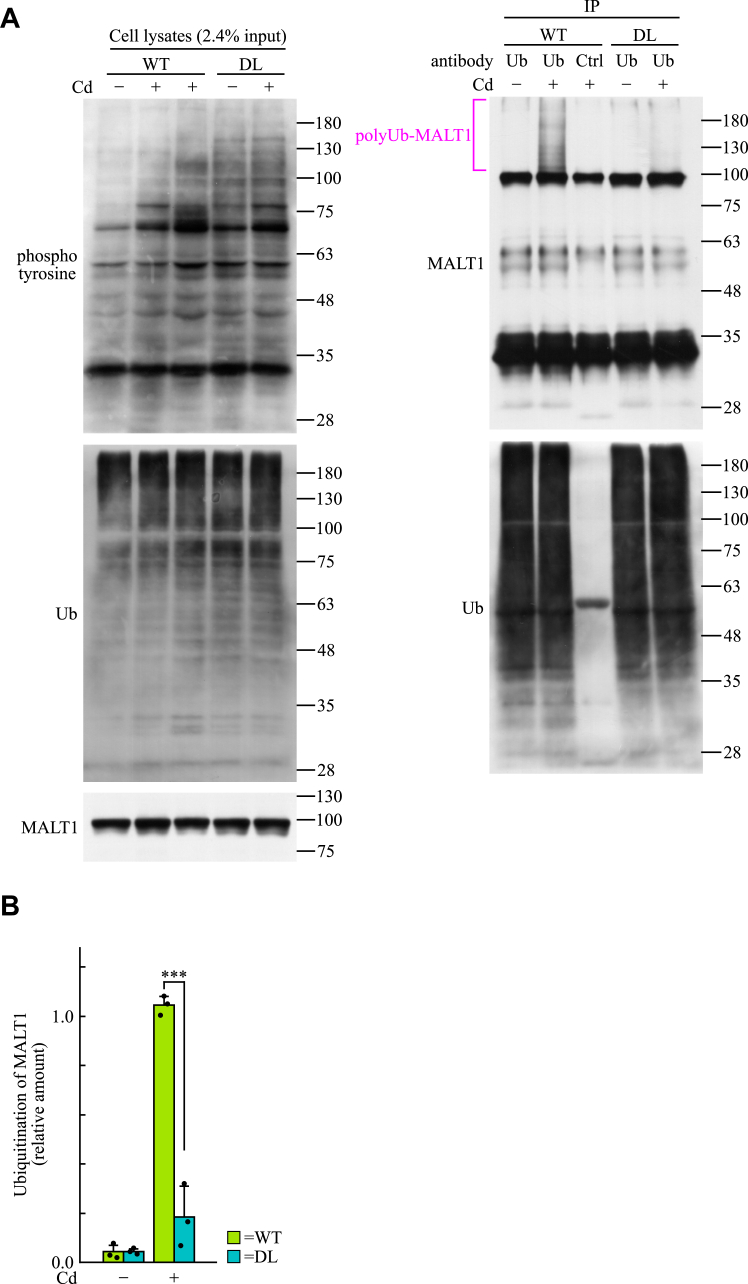
**3BP2 is essential for the ubiquitination of MALT1 in dectin-1–stimulated BMDCs.***A*, BMDCs obtained from WT or 3BP2^DL/DL^ (DL) mice were stimulated without (Cd−) or with (Cd+) curdlan for 30 min. Cell lysates were subjected to immunoprecipitation using anti-ubiquitin (Ub) or control (Ctrl) antibody. The cell lysates and immunoprecipitates (IP) were subjected to immunoblotting analysis. Data are representative of an individual biological replicate of three independent experiments. The bar labeled with polyUb-MALT1 indicates the positions of ubiquitinated MALT1. *B*, relative ubiquitination of MALT1 (relative to MALT1 in cell lysates) in immunoprecipitates was quantified using the immunoblots shown in panel *A*. Closed circles correspond to an individual biological replicate of three independent experiments. Data are presented as the mean ± SD. Statistical significance was analyzed using Student’s *t* test. ∗∗∗*p* < 0.001 was considered statistically significant.

Ubiquitination and activation of MALT1 are essential for dectin-1–induced gene expression in BMDCs (9, 14). Our results suggested that 3BP2 is required for dectin-1–induced gene expression in BMDCs (Fig. 1). Our data also indicated that 3BP2 is involved in the regulation of CARD9-dependent activation of NF-κB and TRAF6-mediated ubiquitination of cellular proteins in HEK-293T cells (Figs. 2 and 3). In view of these observations, we examined whether 3BP2 is required for the ubiquitination of MALT1 in dectin-1–stimulated BMDCs. Immunoblotting analysis showed that ubiquitination of proteins in cell lysates was similar between WT and DL cells, regardless of the stimulation with curdlan (Fig. 5*A*). To analyze the ubiquitination of MALT1, ubiquitinated proteins in cell lysates were immunoprecipitated using anti-ubiquitin antibody, and the immunoprecipitates were subjected to immunoblotting with anti-MALT1 antibody. We observed that MALT1 was dramatically ubiquitinated in WT cells stimulated with curdlan. In contrast, the amount of ubiquitinated MALT1 in DL cells stimulated with curdlan was significantly lower than that in WT cells (Fig. 5, *A* and *B*). These results suggest that 3BP2 is essential for the dectin-1–induced ubiquitination of MALT1, which leads to the CARD9-dependent activation of NF-κB (9, 14).

### 3BP2 is required for optimal activation of MALT1 and is subjected to MALT1-dependent cleavage in dectin-1–stimulated BMDCs

**Figure 6:**
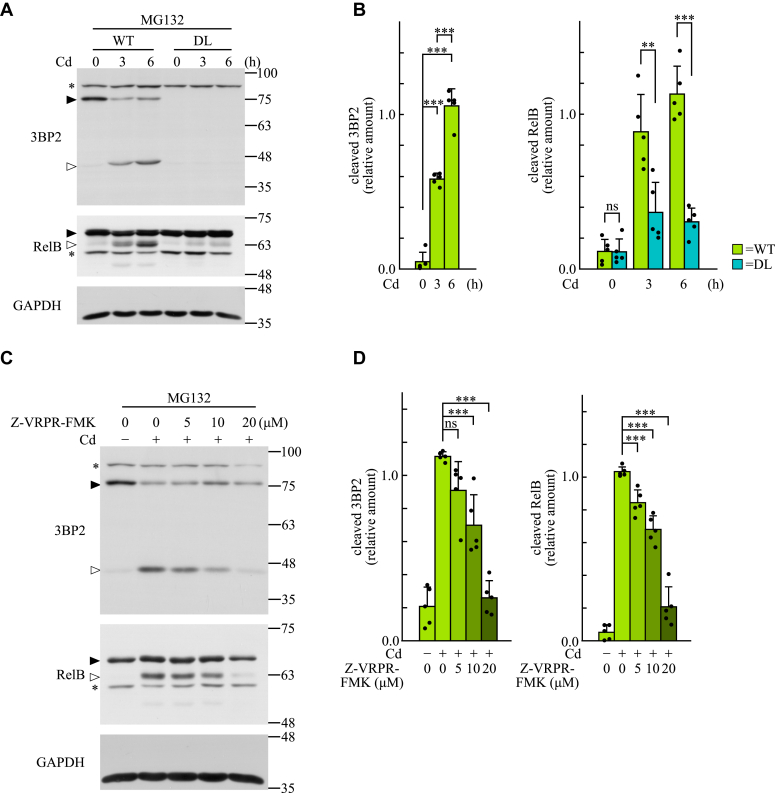
**3BP2 is required for the activation of MALT1 and cleaved in a MALT1-dependent manner in dectin-1–stimulated BMDCs.***A* and *C*, BMDCs obtained from WT mice or 3BP2^DL/DL^ mice (DL) were pretreated with MG132 and stimulated with curdlan (Cd) for the indicated periods of time (*A*), or these cells were stimulated without (Cd−) or with (Cd+) curdlan for 4 h in the presence of the indicated concentrations of MALT1 inhibitor Z-VRPR-FMK (*C*). Cell lysates were subjected to immunoblotting analysis. The positions of the uncleaved and cleaved proteins are indicated by closed and open arrowheads, respectively. ∗ represents the position of a protein nonspecifically recognized by the primary antibody. Data are representative of an individual biological replicate of five independent experiments. *B* and *D*, relative amounts of cleaved 3BP2 or RelB (relative to GAPDH) were quantified using the immunoblots shown in panels *A* and *C*. Closed circles correspond to an individual biological replicate of five independent experiments. Data are presented as the mean ± SD. Statistical significance was analyzed using one-way ANOVA followed by the Tukey–Kramer test (*B*, cleaved 3BP2), Student’s *t* test (*B*, cleaved RelB), or one-way ANOVA followed by Dunnett’s multiple comparison test (*D*). ∗∗∗*p* < 0.001 or ∗∗*p* < 0.01 was considered statistically significant. ns = not significant.

We next examined whether 3BP2 is required for the activation of MALT1 and is cleaved in dectin-1–stimulated BMDCs. MALT1 cleaves RelB, a subunit of NF-κB, in dectin-1–stimulated BMDCs (7, 46). Because pretreatment with the proteasome inhibitor MG132 prevents proteasomal degradation of RelB fragment cleaved by MALT1 and leads to accumulation in cells (7, 47), we pretreated BMDCs with MG132 before stimulation with curdlan. Stimulation with curdlan induced the cleavage of RelB, whereas the amounts of cleaved fragment were significantly lower in DL cells when compared with those in WT cells during the time course (Fig. 6, *A* and *B*). In addition, 3BP2 was cleaved in curdlan-stimulated WT cells, and the amounts of cleaved fragment were gradually increased after stimulation. Taken together, these results suggest that 3BP2 is required for the activation of MALT1 and is subjected to proteolytic cleavage in response to the stimulation of dectin-1. Notably, only one band corresponding to the cleaved fragment of endogenous 3BP2 was detected in curdlan-stimulated BMDCs, whereas two bands (upper and lower) were detected in HEK-293T cells overexpressing HA-tagged human 3BP2 (Fig. 4*A*).

To examine whether MALT1 is responsible for the cleavage of 3BP2, we analyzed the effects of the MALT1 inhibitor, Z-VRPR-FMK (16, 47), on the cleavage of 3BP2 in curdlan-stimulated WT cells. As previously reported (47), MALT1-dependent cleavage of RelB was inhibited by Z-VRPR-FMK treatment in a dose-dependent manner (Fig. 6, *C* and *D*). Under these conditions, we observed that the cleavage of 3BP2 was similarly inhibited. Taken together, these results suggest that 3BP2 is required for the activation of MALT1 and is subjected to MALT1-dependent cleavage in dectin-1–stimulated BMDCs.

### Dectin-1–induced expression of cytokine genes is not influenced by MALT1-dependent cleavage of 3BP2 in BMDCs

**Figure 7:**
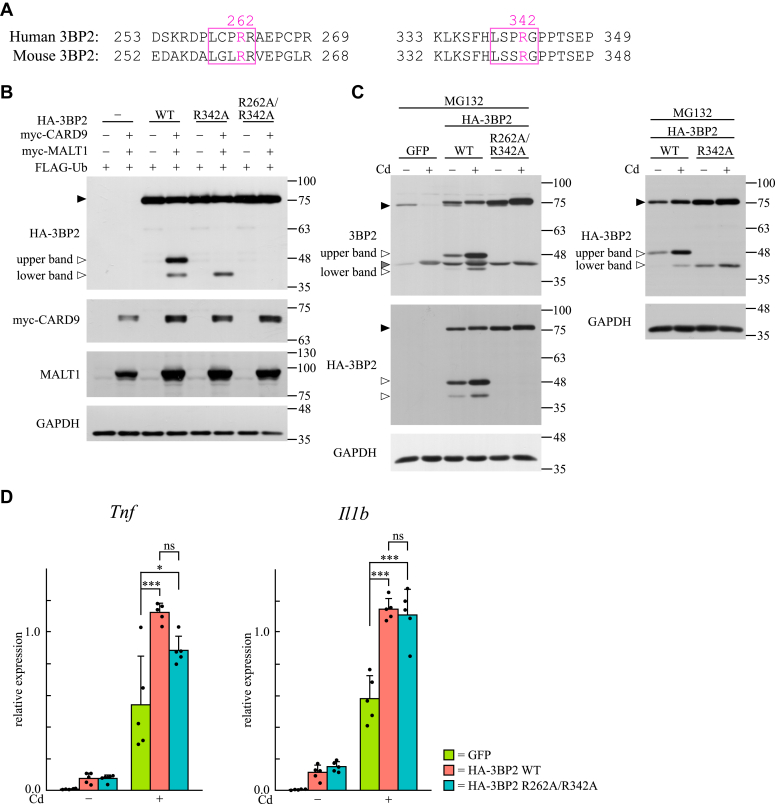
**Dectin-1–induced expression of cytokine genes is not influenced by MALT1-dependent cleavage of 3BP2 in BMDCs.***A*, alignment of amino acid sequences of human and mouse 3BP2. Characters in *red* in boxed sequences are potential sites of cleavage by MALT1. *B*, HEK-293T cells were transfected without (−) or with (+) plasmids encoding the indicated proteins, and the cell lysates were subjected to immunoblotting analysis. The positions of uncleaved or cleaved HA-3BP2 are indicated by closed or open arrowheads (*upper* and *lower* bands), respectively. Data are representative of an individual biological replicate of three independent experiments. *C*, BMDCs obtained from WT mice were infected with lentivirus for exogenous expression of the indicated proteins. Cells were pretreated with MG132 and stimulated without (Cd−) or with (Cd+) curdlan for 4 h. The cell lysates were subjected to immunoblotting analysis. The positions of uncleaved endogenous 3BP2 and HA-3BP2 are indicated by closed arrowheads. The positions of cleaved endogenous 3BP2 or HA-3BP2 are indicated by *gray* or open arrowheads (*upper* and *lower* bands), respectively. Data are representative of an individual biological replicate of three independent experiments. *D*, BMDCs obtained from WT mice were infected with lentivirus for exogenous expression of the indicated proteins. After the cells were stimulated without (Cd−) or with (Cd+) curdlan for 4 h, total RNA was extracted and the mRNA expression of cytokines was analyzed using qPCR. Closed circles correspond to an individual biological replicate of five independent experiments. Data are presented as the mean ± SD. Statistical significance was analyzed using one-way ANOVA followed by the Tukey–Kramer test. ∗∗∗*p* < 0.001 or ∗*p* < 0.05 was considered statistically significant. ns = not significant.

MALT1 acts as an Arg-specific protease, and its substrate specificity has been extensively investigated (16, 44). L-X-S-R-G or L-X-P-R-G (X represents an undefined amino acid) motifs serve as recognition sites for MALT1 (48). Based on this report, we identified two sites that are potentially recognized by MALT1 in 3BP2 (Fig. 7*A*). One of the cleavage sites is Arg342 in human 3BP2 (Arg341 in mouse 3BP2), which is located in a sequence that completely matches the motif. The other site is Arg262 in human 3BP2 (Arg261 in mouse 3BP2), which is located in a sequence similar to but not matching the motif. To examine whether Arg342 and/or Arg262 in human 3BP2 are cleaved in a MALT1-dependent manner, HEK-293T cells were transfected with plasmids encoding HA-tagged R342A or R262A/R342A mutants of human 3BP2 together with those encoding CARD9, MALT1, and FLAG-tagged ubiquitin. As shown in Figure 4*A*, the upper and lower bands corresponding to the cleaved fragment of HA-tagged 3BP2 were detected in the lysates of cells expressing HA-tagged 3BP2 together with CARD9, MALT1, and FLAG-tagged ubiquitin (Fig. 7*B*, lane 4). However, the lower band, but not the upper band, was detected in the lysates of cells expressing the R342A mutant (lane 6). Furthermore, no band was detected in the lysates of cells expressing the R262A/R342A mutant (lane 8). These results suggested that Arg342 and Arg262 in human 3BP2 are the major cleavage sites for CARD9-activated MALT1 in HEK-293T cells.

Next, we examined whether Arg342 and Arg262 in human 3BP2 is subjected to MALT1-dependent cleavage in curdlan-stimulated BMDCs. Lentivirus-infected WT cells expressing GFP, HA-tagged 3BP2, R262A/R342A mutant, or R342A mutant were stimulated with curdlan, and cell lysates were subjected to immunoblotting analysis. In the lysates of cells expressing HA-tagged 3BP2, we detected upper and lower bands, in addition to the band corresponding to cleaved fragments of endogenous 3BP2 (Fig. 7*C*, left panel). The levels of these three proteins increased in response to curdlan stimulation (lane 3 vs. 4). Immunoblotting analysis using anti-HA antibody confirmed that the upper and lower bands were derived from HA-tagged 3BP2. Conversely, in the lysates of cells expressing the HA-tagged R262A/R342A mutant, neither the upper nor lower bands could be detected. In addition, in the lysates of cells expressing the R342A mutant, the lower bands, but not the upper bands, were detected using anti-HA antibody (Fig. 7*C*, right panel). These results demonstrated that Arg342 and Arg262 in human 3BP2 are the major cleavage sites of MALT1 in dectin-1–stimulated BMDCs.

Finally, we examined whether the cleavage of 3BP2 affects dectin-1–induced expression of cytokine mRNAs (Fig. 7*D*). The mRNA expression of TNF-α and IL-1β was increased in WT cells expressing GFP upon stimulation with curdlan, and it was further upregulated by the expression of HA-tagged 3BP2 instead of GFP. In cells expressing the R262A/R342A mutant, the expression of cytokine mRNAs was upregulated in response to stimulation with curdlan, and the expression levels of these mRNAs were comparable to those observed in cells expressing HA-tagged WT 3BP2. Similar results were obtained using a different dose of curdlan and different treatment duration (Fig. S2). These results suggest that the dectin-1–induced expression of cytokines is not influenced by the MALT1-dependent cleavage of 3BP2.

### Identification of HS1 as an interacting molecule with the SH2 domain of 3BP2 in dectin-1–stimulated BMDCs

**Figure 8:**
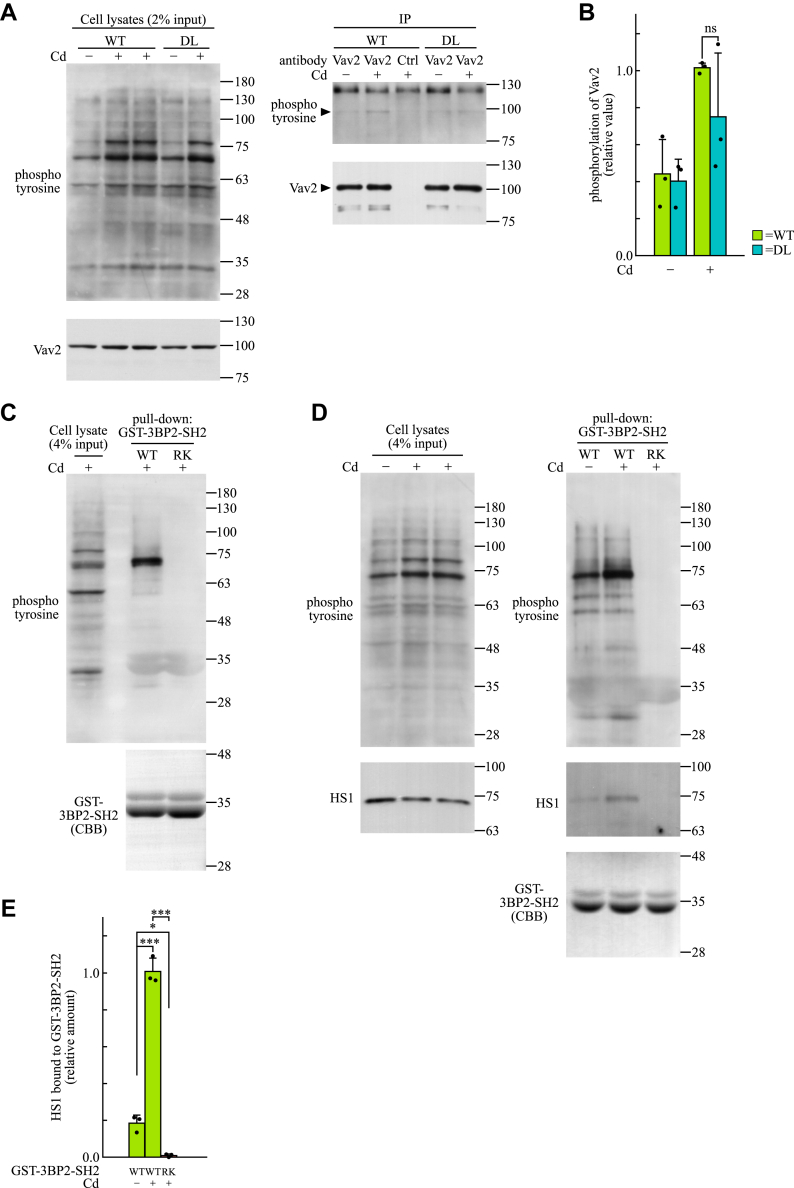

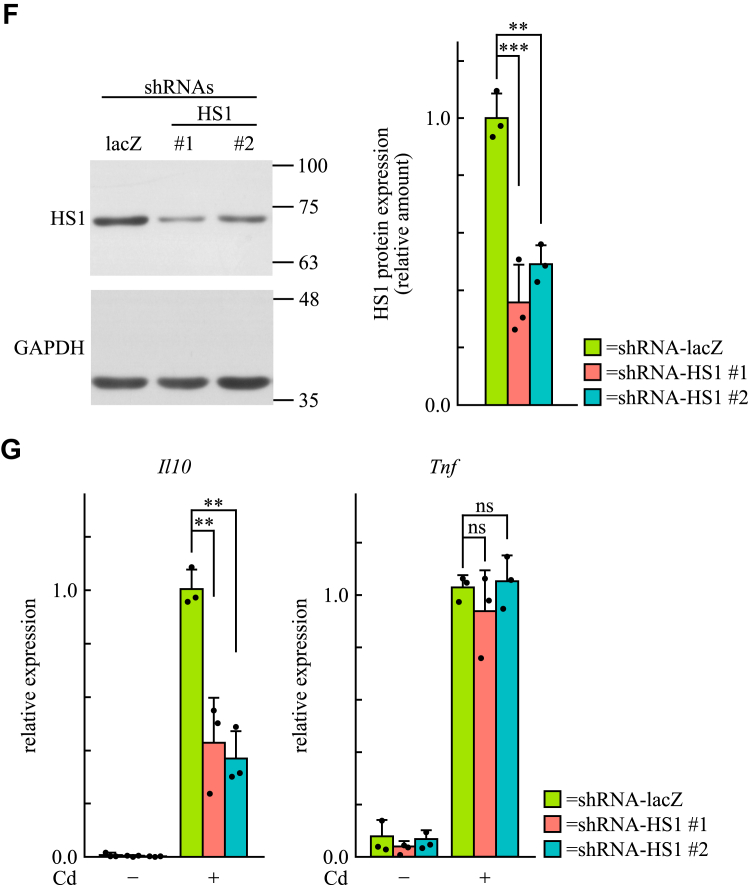
**Identification of HS1 as a target of 3BP2 in dectin-1–stimulated BMDCs.***A*, BMDCs obtained from WT or 3BP2^DL/DL^ (DL) mice were stimulated without (Cd−) or with (Cd+) curdlan for 30 min. Cell lysates were subjected to immunoprecipitation using anti-Vav2 (Vav2) or control (Ctrl) antibody. The cell lysates and immunoprecipitates (IP) were subjected to immunoblotting analysis. The positions of Vav2 are indicated using closed arrowheads. Data are representative of an individual biological replicate of three independent experiments. *B*, relative phosphorylation of Vav2 (relative to immunoprecipitated Vav2) was quantified using the immunoblots shown in panel A. Closed circles correspond to an individual biological replicate of three independent experiments. Data are presented as the mean ± SD. *C* and *D*, BMDCs obtained from WT mice were stimulated without (Cd−) or with (Cd+) curdlan for 30 min. Cell lysates were subjected to pull-down assay using GST-3BP2-SH2-WT (WT) or -R486 K (RK). The cell lysates and co-precipitated proteins (pull-down) were subjected to immunoblotting analysis. The amount of GST-3BP2-SH2 bound to GSH beads was confirmed using Coomassie Brilliant Blue staining (CBB). Data are representative of an individual biological replicate of three independent experiments. *C*, cell lysate obtained from curdlan-stimulated BMDCs (Cd+) was equally divided into two. Each cell lysate was used for pull-down assay using the indicated protein. The coprecipitated proteins were subjected to the DIA-MS analysis. *E*, relative amount of HS1 bound to GST-3BP2-SH2 (relative to HS1 in cell lysates) was quantified using the immunoblots shown in panel *D*. Closed circles correspond to an individual biological replicate of three independent experiments. Data are presented as the mean ± SD. *F* and *G*, BMDCs obtained from WT mice were infected with lentivirus for expression of the indicated shRNAs. *F*, the cell lysates were subjected to immunoblotting analysis to assess HS1 knockdown. *Left* panels: representative immunoblots of an individual biological replicate from three independent experiments. *Right* panel: relative amounts of HS1 (relative to GAPDH) quantified using the immunoblots shown in the *left* panels. Closed circles correspond to an individual biological replicate of three independent experiments. Data are presented as the mean ± SD. *G*, after stimulation without (Cd−) or with (Cd+) curdlan for 4 h, total RNA was extracted, and the mRNA expression of indicated genes was analyzed using qPCR. Closed circles correspond to an individual biological replicate of three independent experiments. Data are presented as the mean ± SD. Statistical significance was analyzed using Welch’s *t* test (*B*), two-way ANOVA followed by the Tukey–Kramer test (*E*), one-way ANOVA followed by Dunnett’s multiple comparison test (*F* and *G*). ∗∗∗*p* < 0.001, ∗∗*p* < 0.01, or ∗*p* < 0.05 was considered statistically significant. ns = not significant.

Given that Vav family proteins play important roles on the regulation of MALT1 through CARD9 (7), we examined whether 3BP2 regulates the activation of Vav family proteins in dectin-1–stimulated BMDCs. Because the activation of Vav family proteins is tightly regulated by the phosphorylation of tyrosine residues (49), Vav2 was immunoprecipitated from cell lysates and the tyrosine phosphorylation was evaluated using immunoblotting analysis. The tyrosine phosphorylation of Vav2 was induced by the stimulation with curdlan, although the level of phosphorylation was comparable between WT and DL cells (Fig. 8, *A* and *B*). These results indicated that 3BP2 is not essential for the activation of Vav2 in dectin-1–stimulated BMDCs. These results also indicated that 3BP2 regulates the gene expression and functions of MALT1 through a Vav family protein-independent mechanism.

Our previous study suggested that the SH2 domain of 3BP2 is critical for the CARD9-mediated activation of NF-κB (28). To understand the mechanism how 3BP2 regulates the dectin-1–mediated signaling, we performed pull-down assay to identify proteins interacting with the SH2 domain of 3BP2 in dectin-1–stimulated BMDCs (Fig. 8*C*). We observed that a tyrosine-phosphorylated protein, with a molecular weight of 75 kDa, coprecipitated with glutathione *S*-transferase (GST) fused with the SH2 domain of WT 3BP2 (GST-3BP2-SH2-WT) but not with the R486K mutant (GST-3BP2-SH2-RK). This might be because the mutation causes a loss of function of the SH2 domain (19). To identify the protein, an equal amount of cell lysate proteins was subjected to pull-down assay using GST-3BP2-SH2-WT or GST-3BP2-SH2-RK, and the coprecipitated proteins were analyzed using data-independent acquisition (DIA)-mass spectrometry (MS). A total of 5818 proteins were identified as coprecipitated proteins with GST-3BP2-SH2 (Table S3). Among these, HS1 encoded by the *Hcls1* gene was highly enriched in precipitates with GST-3BP2-SH2-WT when compared with that precipitated with GST-3BP2-SH2-RK (fold change of 1173.35). To confirm whether HS1 interacts with the SH2 domain of 3BP2, we performed pull-down assay using cell lysates prepared from WT cells. We observed that HS1 was co-precipitated with GST-3BP2-SH2-WT but not with GST-3BP2-SH2-RK, and the amount of HS1 co-precipitated with GST-3BP2-SH2-WT was increased upon stimulation with curdlan (Fig. 8, *D* and *E*).

To determine whether HS1 is involved in dectin-1–induced cytokine gene expression in BMDCs, we conducted knockdown experiments using shRNAs. HS1 protein levels were significantly lower in BMDCs expressing shRNAs targeting *Hcls1* (shRNA-HS1 #1 or #2) than in those expressing control shRNA targeting *lacZ* in *E. coli* (shRNA-lacZ) (Fig. 8*F*). In addition, the dectin-1–induced expression of *Il10* was dramatically lower in BMDCs expressing shRNA-HS1 #1 or #2 than in those expressing shRNA-lacZ (Fig. 8*G*). Notably, the dectin-1–induced expression of *Tnf* was not significantly reduced by the expression of shRNAs targeting *Hcls1*. These results suggest that 3BP2 regulates dectin-1–induced gene expression, at least in part, through its interaction with HS1 in BMDCs.

## Discussion

We previously reported that 3BP2 is required for the dectin-1–induced mRNA expression of TNF-α, IL-1β, IL-2, IL-6, IL-10, and IL-23p19 in BMDCs (28). The CARD9-dependent production of these cytokines is believed to play an important role in antifungal immunity (9, 10). In this study, using microarray analysis, we found that 3BP2 is also required for dectin-1–induced expression of *Nos2* and *Ptgs2* (Fig. 1). The induction of inducible nitric oxide synthase encoded by *Nos2* may lead to an increase in the cellular levels of reactive oxygen species (50). As we showed that 3BP2 is involved in the Fc receptor-mediated phagocytosis of opsonized zymosan (yeast cell walls) in interferon-gamma-primed U937 cells (21), it is likely that 3BP2 plays an important role in the removal of pathogenic fungi *via* phagocytes. In addition, the induction of cyclooxygenase-2 encoded by *Ptgs2* might increase the release of arachidonic acid metabolites, which exacerbate inflammation (51). Therefore, 3BP2 may contribute to dectin-1–induced immune responses through the induction of these genes, in addition to those of cytokines. Interestingly, we found that the expression of *Ereg* was induced in dectin-1–stimulated BMDCs and that 3BP2 is involved in this regulation. Epiregulin, encoded by *Ereg*, acts as a ligand for the epidermal growth factor receptor. Importantly, epidermal growth factor receptor kinase-defective *waved-2* and *Ereg*-deficient mice showed increased susceptibility to DSS-induced colitis (38, 52). Genome-wide association studies have shown that the genetic variants of human *TNFSF15* are associated with inflammatory bowel disease (53). In addition, the TNF-like ligand 1A encoded by *Tnfsf15* plays an important role in mucosal healing in DSS-induced colitis (39). Importantly, *CARD9* is also associated with inflammatory bowel disease (53), and studies using *Card9*-deficeint mice showed that CARD9 has protective roles against DSS-induced colitis (40, 41). Collectively, our study raises the possibility that 3BP2 is involved in the regulation of mucosal immunity by inducing a variety of genes, including *Ereg* and *Tnfsf15*, *via* CARD9.

Formation of molecular complexes with BCL10 and MALT1 is essential for CARD9 to induce the activation of NF-κB (9, 14). We found that both BCL10 and MALT1, in addition to CARD9, were essential for the 3BP2-medaited activation of NF-κB in HEK-293T cells (Fig. 2). Therefore, overexpression of 3BP2 may lead CARD9 to form molecular complexes with BCL10 and MALT1 endogenously expressed in HEK-293T cells. In addition to BCL10 and MALT1, TRAF6 is believed to play important roles in the dectin-1–induced activation of NF-κB through ubiquitination of cellular proteins such as MALT1 (9, 13, 15). Because our data suggest that the signaling pathway mediated by TRAF6 overlaps with that induced by the overexpression of 3BP2 with CARD9 (Fig. 2), it is possible that 3BP2 is involved in the regulation of TRAF6-dependent ubiquitination of MALT1 through CARD9.

In this study, we show that the overexpression of 3BP2 induced the ubiquitination of cellular proteins *via* CARD9 in HEK-293T cells (Fig. 3). It seems likely that the 3BP2-mediated ubiquitination of cellular proteins and activation of NF-κB is tightly coupled with each other because the following phenomena were commonly observed: i) These were not influenced by the mutation of Thr231 to Ala, but by the mutation of Lys125 to Arg in CARD9 (Figs. 3 and S4); ii) these were required for BCL10, MALT1, and TRAF6 (Figs. 2 and 3); iii) these effects are not enhanced by TRAF6 overexpression (Figs. 2 and 3). Collectively, these findings support our hypothesis that the overexpression of 3BP2 activates TRAF6 through CARD9, which leads to the activation of NF-κB. In these experiments, we found that overexpression of 3BP2 induced MALT1-dependent cleavage of 3BP2 in a CARD9-dependent manner (Figs. 3 and 4). It was reported that the expression level of MALT1 is reduced by TRAF6 overexpression in HEK-293T cells (54). This observation might account for our data indicating that the amounts of the cleaved 3BP2 fragment were slightly reduced by the overexpression of TRAF6 (Fig. 3*D*, lane 2 vs. 4, and lane 6 vs. 8). Surprisingly, we found that exogenous expression of BCL10 in BCL10-KO cells rescued 3BP2-mediated activation of NF-κB (Fig. 2) and ubiquitination of cellular proteins, but not the cleavage of 3BP2 (Fig. S1, lane 6 vs. 7). Although we cannot provide a possible explanation for this phenomenon, we found that the cleavage of 3BP2 was dramatically reduced in parental cells overexpressing BCL10 (Fig. S1, lane 2 vs. 3). Therefore, we hypothesized that the protease activity of endogenous MALT1 was influenced by the expression level of BCL10. Regarding the protease activity of MALT1, overexpression of FLAG-tagged ubiquitin resulted in an increase in the levels of the cleaved fragment of 3BP2 (Fig. 4). This might be because the overexpression of FLAG-tagged ubiquitin accelerates the monoubiquitination of MALT1 by an unidentified enzyme, which in turn enhances the MALT1 protease activity, as previously reported (14, 45).

In this study, we obtained data indicating that 3BP2 is required for the ubiquitination of MALT1 in dectin-1–stimulated BMDCs (Fig. 5). Stimulation of dectin-1 accelerates the formation of molecular complexes of BCL10, MALT1, and CARD9, which is required for the ubiquitination of MALT1 to activate NF-κB through IKK α and β (13, 14, 15). In a previous study, we showed that the dectin-1–induced phosphorylation of IKK α and β was dramatically diminished in DL cells (28). Therefore, it is reasonable to consider that 3BP2 is required for the dectin-1–induced formation of molecular complexes, thereby regulating the ubiquitination of MALT1. This is supported by the data suggesting that 3BP2 is also required for the protease activity of MALT1 in dectin-1–stimulated BMDCs (Fig. 6) because the protease activity of MALT1 is also regulated by the formation of molecular complex with BCL10 and CARD9 (9, 14, 16, 44).

Activated MALT1 cleaves several signaling molecules, such as BCL10, MALT1, and RelB (14, 16, 47, 55). The present study indicates that 3BP2 is also subjected to MALT1-dependent cleavage in dectin-1–stimulated BMDCs (Figs. 6 and 7). Although we found that Arg262 and Arg342 in human 3BP2 are the major cleavage sites for MALT1, it seems that MALT1 cleaves only one site, presumably Arg341 in mouse 3BP2, in dectin-1–stimulated BMDCs. This may be because the amino acid sequence around Arg261 in mouse 3BP2 is not as conserved as that in human 3BP2 for recognition by MALT1. In these experiments, we found that overexpression of human 3BP2 resulted in increased levels of cleaved 3BP2, even in nonstimulated BMDCs (Fig. 7*C*). Therefore, it is likely that overexpression of 3BP2 enhances the basal activity of MALT1 in nonstimulated BMDCs, as observed in HEK-293T cells. Importantly, the 3BP2 protein dramatically accumulated in bone marrow-derived osteoclasts obtained from cherubism mutant mice, which leads to constitutive activation of the signaling molecules Src, Syk, and Vav (27). Based on our observations, it is possible that the CARD9-mediated immune response is constitutively activated in the macrophages and dendritic cells of cherubism-mutant mice, thereby contributing to the development of the pathology. Regarding the cleavage of 3BP2, it appears that the dectin-1–induced expression of *Tnf* and *Il1b* was not influenced by the cleavage of 3BP2 (Figs. 7*D* and S2). Based on studies using electron microscopy, it is thought that CARD9 induces oligomerization and filament formation of BCL10 accompanied with the activation of MALT1 after the formation of the molecular complexes with BCL10 and MALT1 (56). The BCL10-MALT1 filament assembly amplifies the cellular signaling leading to the activation of NF-κB (14, 56). Therefore, we would like to propose that 3BP2 is involved in the dectin-1–induced formation of molecular complexes to activate MALT1 but is not required for the late signaling events that facilitate the BCL10-MALT1 filament assembly for propagating the activation of NF-κB.

CARD9 is essential for the dectin-1–induced expression of cytokine genes through NF-κB activation, and Vav family proteins in addition to PKCδ participate in the regulation (6, 7, 9). Given that 3BP2 regulates the functions of Vav family proteins in antigen-stimulated cells (19, 20, 57), Vav2 may also act as a downstream effector of 3BP2 in the activation of NF-κB. Notably, our results suggested that endogenous Vav2 in HEK-293T cells is activated by the overexpression of 3BP2 (Fig. S3). However, Vav2 knockdown had no effect on the 3BP2-mediated activation of NF-κB in HEK-293T cells, although overexpression of the dominant active Vav2 mutant increased the basal level of NF-κB reporter activity (Fig. S4). Furthermore, our results indicated that 3BP2 is not essential for the activation of Vav family proteins in dectin-1–stimulated BMDCs (Fig. 8). Taken together, Vav2 may not act as a downstream target of 3BP2; rather, it may have the potential to stimulate 3BP2-mediated activation of NF-κB downstream of CARD9. Regarding PKCδ, treatment with a pan-PKC inhibitor, sotrastaurin, as well as overexpression of the dominant negative PKCδ mutant, had no effect on the 3BP2-mediated activation of NF-κB in HEK-293T cells. However, overexpression of the dominant active PKCδ mutant increased NF-κB reporter activity (Fig. S4). These findings suggest that PKCδ is not a downstream effector of 3BP2 in HEK-293T cells, consistent with our observations in dectin-1–stimulated BMDCs (28). Instead, our results suggested PKCδ activation enhances 3BP2-mediated signaling *via* the phosphorylation of CARD9 (Fig. S4).

To elucidate how 3BP2 regulates dectin-1–mediated signaling, we identified HS1 as a target of 3BP2 in dectin-1–stimulated BMDCs (Fig. 8). The interaction between 3BP2 and HS1 has also been confirmed through interactome analysis using yeast two-hybrid systems (58). HS1 is an adaptor protein essential for the gene expression of IL-2 and the accumulation of filamentous actin at the immune synapse following T-cell receptor ligation (59). In activated lymphocytes, HS1 undergoes tyrosine phosphorylation (59, 60, 61), which is crucial for its localization to the immune synapse and for its interactions with multiple signaling molecules, including Vav family proteins (59). Our results indicated that the level of tyrosine-phosphorylated HS1 bound to the SH2 domain of 3BP2 increased following dectin-1 stimulation (Fig. 8). Notably, knockdown experiments revealed that HS1 is required for the dectin-1–induced expression of *Il10* but not for *Tnf* expression, although the expression of both cytokines was significantly reduced in dectin-1–stimulated DL cells (Fig. 1) (28). These results suggest that 3BP2-mediated signaling in dectin-1–stimulated BMDCs is, at least in part, regulated by HS1. In addition, these results suggest that other signaling molecules may work in conjunction with HS1 to facilitate dectin-1–mediated signaling through 3BP2. Further studies are required to elucidate the molecular mechanisms underlying the regulation of dectin-1–mediated signaling by 3BP2.

**Figure 9:**
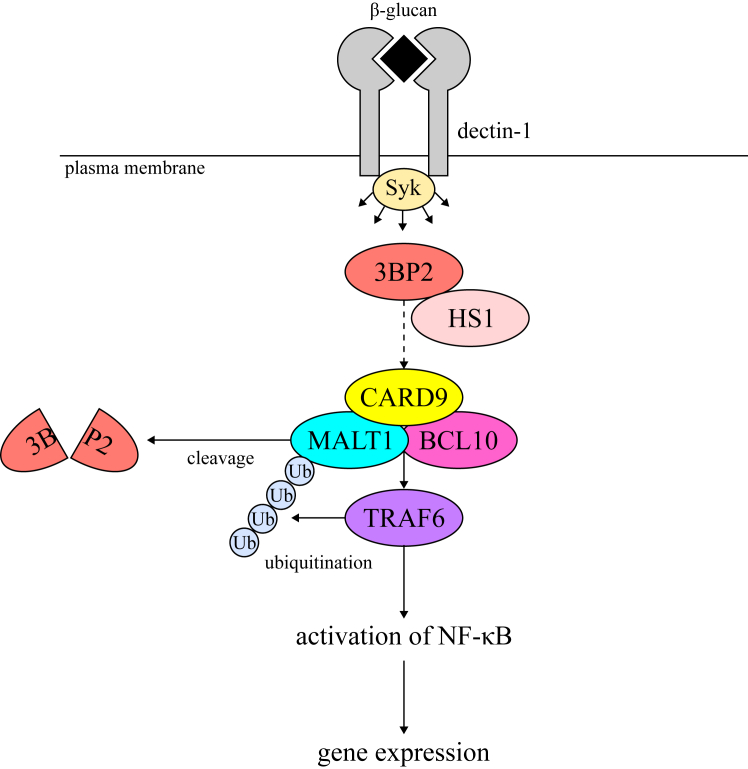
**Schematic representation of regulatory mechanism of dectin-1–induced cellular signaling through 3BP2 proposed in this study.** Stimulation of dectin-1 with β-glucan induces activation of tyrosine kinase Syk, which propagates cellular signaling leading to the CARD9-mediated activation of NF-κB. 3BP2 is required for TRAF6-mediated ubiquitination (Ub) of MALT1 and for acceleration of the protease activity of MALT1. The ubiquitination of MALT1 leads to the activation of NF-κB, which is required for the dectin-1–induced gene expression. Acceleration of protease activity of MALT1 leads to the cleavage of 3BP2. 3BP2 regulates the dectin-1–mediated signaling, at least in part, through its interaction with HS1 (*dotted line*).

In conclusion, we demonstrate that 3BP2 is required for dectin-1–induced gene expression and functions of MALT1. Apparently, 3BP2 regulates the ubiquitination and proteolytic activity of MALT1 through CARD9, at least in part, *via* its interaction with HS1 (Fig. 9). Further studies are required to clarify the role of 3BP2 in dectin-1–mediated signaling. In addition, it is important to analyze the role of 3BP2 in intestinal mucosal immunity to further understand the relationship between 3BP2 and CARD9.

## Experimental procedures

### Mice

3BP2^DL/DL^ mice (28) were backcrossed with C57BL/6J mice (Japan Slc). Mice were used in this study after being euthanized by carbon dioxide inhalation. All the mice were maintained in the Division of Laboratory Animal Resources at the University of Fukui under specific pathogen-free conditions. All animal experiments described in this study were performed in accordance with the Regulations for Animal Research of the University of Fukui and approved by the Animal Research Committee of the University of Fukui.

### Antibodies

Anti-Vav2 (clone EP1067Y, #ab52640) and anti-MALT1 (clone EPR24445-129, #ab283573) antibodies were purchased from Abcam. Anti-PKCδ (clone D10E2, #9616), anti-MALT1 (#2494), anti-TRAF6 (clone D21G3, #8028), anti-RelB (clone D7D7W, #10544), and control (clone DA1E, #3900) antibodies were purchased from Cell Signaling Technology. Anti-myc (clone 10D11, #013-26513) antibody was purchased from Fujifilm Wako Pure Chemicals. Anti-GAPDH (clone 6C5, #MAB374), anti-FLAG (clone M2, #F1804), and anti-phosphotyrosine (clone 4G10, #05-321) antibodies were purchased from Merck Millipore. Anti-HA (clone 16B12, #901514) antibody was purchased from BioLegend. Anti-3BP2 (clone C-11, #sc-377020) and anti-BCL10 (clone 331.3, #sc-5273) antibodies were purchased from Santa Cruz Biotechnology. Anti-ubiquitin (clone FK2, #D058-3) and anti-HS1 (clone 3A3, #M001-3) antibodies were purchased from Medical & Biological Laboratories. For immunoblotting analysis, anti-Vav2, anti-FLAG, anti-RelB, anti-GAPDH, and anti-phosphotyrosine antibodies were used at 1/2000 dilution. Other antibodies were used at 1/1000 dilution. Peroxidase-conjugated goat anti-mouse IgG (#115-035-003) and goat anti-rabbit IgG (#111–035–144) were purchased from Jackson ImmunoResearch. Secondary antibodies were used at 1/10,000 dilution.

### Plasmid DNAs and siRNAs

Plasmids encoding HA-tagged WT human 3BP2 and mutants lacking the PH (3BP2-dPH) or SH2 (3BP2-dSH2) domain were previously described (28). Plasmids encoding FLAG-tagged ubiquitin, pcDNA3.1-myc, and pcDNA3.1-HA were gifts from Dr Keiji Tanaka (Tokyo Metropolitan Institute of Medical Science). The complementary DNA (cDNA) encoding mouse CARD9 in pcDNA3.1-FLAG CARD9 (28) was transferred to the pcDNA3.1-myc vector. cDNAs encoding mouse BCL10, MALT1, TRAF6, DA Vav2 (dominant active form of Vav2; amino acid residues 187–570) (62), PKCδ, and DA PKCδ (dominant active form of PKCδ; amino acid residues 334–674) (63) were amplified using PCR and subcloned in pcDNA3.1-myc, pcDNA3.1-HA, or pEF1/myc-His vectors (ThermoFisher Scientific). cDNAs encoding DN PKCδ (PKCδ K376R) (63), CARD9 T231A, CARD9 K125R, human 3BP2 R342A, and human 3BP2 R262A/R342A were generated by site-directed mutagenesis. The sequences of DNA amplified using PCR were verified *via* DNA sequencing. pNF-κB-luc and phRL-TK were purchased from Clontech and Promega, respectively. px330 and pENTR-Puro were gifted by Dr Feng Zhang (Addgene plasmid #42230) and Dr Tomohiro Kurosaki (Osaka University), respectively. pLenti-CMV-GFP-Puro (658-5) was a gift from Dr Eric Campeau and Dr Paul Kaufman (Addgene plasmid #17448). The cDNA encoding GFP in pLenti-CMV-GFP-Puro (658-5) was replaced with that of the HA-tagged WT or mutant forms of human 3BP2 to obtain pLenti-CMV-HA-3BP2-Puro, pLenti-CMV-HA-3BP2 R342A-Puro, and pLenti-CMV-HA-3BP2 R262A/R342A-Puro. psPAX2 was gifted by Dr Didier Trono (Addgene plasmid #12260). The plasmids pLKO.1-puro and pCMV-VSV-G were generously provided by Dr Bob Weinberg (Addgene plasmid #8453 and #8454, respectively) (64). The target sequences for shRNAs were provided by Broad Institute (https://www.broadinstitute.org/), and oligo DNAs designed for shRNA expression were inserted into the *Age*I-*EcoR*I sites of pLKO.1-puro. The specific targets for shRNAs are as follows: pLKO.1-puro shRNA-lacZ, 5′-CCCGTCAGTATCGGCGGAATT-3′; pLKO.1-puro shRNA-HS1 #1, 5′-GAGCGAAAGGCTGTGGTAAAG-3′; and pLKO.1-puro shRNA-HS1 #2, 5′-CCCGCCTCTTTAAGAGCTTTA-3′. pGEX-4T-3-3BP2-SH2-WT and -R486K were described previously (19). Predesigned control (#4390843), Vav2 #1 (ID: 13196), and Vav2 #2 (ID: s14755) siRNAs were purchased from Thermo Fisher Scientific.

### Cell culture, transfection, and treatment

Cells were maintained in a humidified atmosphere with 5% CO_2_ at 37 °C. HEK-293T cells were provided by Dr Shota Yamauchi (University of Tokyo) and cultured in Dulbecco’s modified Eagle’s medium (Fujifilm Wako Pure Chemicals) supplemented with 10% heat-inactivated fetal bovine serum (MP Biomedicals), penicillin, and streptomycin. Transfection of plasmid DNA was performed using the X-tremeGENE9 DNA transfection reagent (Merck Millipore) according to the manufacturer’s instructions. Transfection of siRNA (20 nM) was performed using Lipofectamine RNAiMAX (Thermo Fisher Scientific) according to the manufacturer’s instructions. At 48 h after siRNA transfection, cells were transfected with plasmid DNA. To inhibit the enzymatic activity of endogenous PKCδ, HEK-293T cells were cultured with a selective pan-PKC inhibitor, sotrastaurin (Selleck Chemicals) after transfection. To obtain BMDCs, bone marrow cells were collected from the tibia and femur of mice using a 1 ml syringe with a 26-gauge needle and cultured in Dulbecco’s modified Eagle’s medium supplemented with 10% fetal bovine serum, penicillin, streptomycin, and 40 ng/ml recombinant granulocyte-macrophage colony-stimulating factor (Fujifilm Wako Pure Chemicals). On day 6, the bulk cultures were harvested and were used for the experiments. To inhibit proteasome activity, BMDCs were treated with MG132 (10 μM, Fujifilm Wako Pure Chemicals) for 30 min (47). The cells were then centrifuged, and the growth medium was replaced with fresh medium to remove MG132. To inhibit the protease activity of MALT1, BMDCs were stimulated in the presence of Z-VRPR-FMK (AdipoGen Life Sciences).

### Stimulation with curdlan

Tissue culture plates were coated with curdlan as described previously, with minor modification (28). In brief, tissue culture plates were coated with 300 μg/ml curdlan (Fujifilm Wako Pure Chemicals) solubilized in 0.2 M NaOH overnight at 4 °C. After unbound curdlan was removed, the plates were washed once with distilled water and air-dried. The BMDCs were seeded into curdlan-coated wells.

### Microarray analysis

BMDCs were stimulated with curdlan for 4 h. After washing with PBS, the total RNA was extracted and purified using acid guanidinium thiocyanate-phenol-chloroform extraction (65) and treated with DNase I (Fujifilm Wako Pure Chemicals) at 25 °C for 15 min, followed by phenol-chloroform extraction. The quality of the purified RNA was analyzed using the Agilent 2100 Bioanalyzer (Agilent Technologies), and RNA integrity numbers were estimated at 9.0 and above. The RNA was subjected to the Clariom S assay, and gene expression arrays were analyzed using the GeneChip system GCS3000 (Thermo Fisher Scientific). The raw data (CEL files) were processed with the SST-RMA algorithm using Transcriptome Analysis Console software version 4.03 (Thermo Fisher Scientific) for background adjustment, intensity normalization, probe set summarization, and log2 transformation of the data. We used the absolute value of log2ratio >1 and *p*-value <0.05 as the threshold to judge the significance of the difference in gene expression. Venn diagram, a volcano plot, and the result of hierarchical clustering analysis were also prepared using the Transcriptome Analysis Console software.

### Preparation of cell lysate and immunoblotting

Cells were washed with PBS and lysed with lysis buffer (50 mM Tris–HCl, pH 7.4, 150 mM NaCl, 100 mM NaF, 0.1 mM Na_3_VO_4_, 10 mM EDTA, 1% Triton-X 100, and 2 μg/ml aprotinin) as described previously (19). Insoluble materials were removed from cell lysates by centrifugation at 17,700*g* for 10 min at 4 °C. The protein concentration in the supernatant was estimated using the BCA protein assay kit (Fujifilm Wako Pure Chemicals) and normalized between the samples. After the addition of SDS sampling buffer, cell lysates were incubated at 95 °C for 5 min, separated using SDS-PAGE, and analyzed using immunoblotting, as described previously (19). Equal amounts of protein were loaded in each lane. Quantification of immunoblots was performed using densitometry with the ImageJ software (National Institutes of Health) and normalized against GAPDH, as described previously (28).

### Luciferase assay

HEK-293T cells were transfected with various expression plasmids together with pNF-κB-luc and phRL-TK. Twenty hours after transfection, a luciferase assay was performed using the PicaGene Luminescence Kit (TOYO B-NET) according to the manufacturer’s instructions.

### Generation of BCL10-KO, MALT1-KO, or TRAF6-KO cells

To express the CRISPR/Cas9 system in HEK-293T cells, complementary oligo DNAs for the expression of single-guide RNAs corresponding to each target gene were annealed and ligated into a *Bbs*I-digested px330 vector. Guide RNA sequences were designed using the Invitrogen TrueDesign Genome Editor tool (Thermo Fisher Scientific). The sequences of the ligated oligonucleotide DNAs were verified using DNA sequencing. Each px330 designed for the target genes was transfected into HEK-293T cells together with pENTR-Puro. Transfected cells were grown in the presence of puromycin (2 μg/ml, InvivoGen) for 2 to 3 weeks. Puromycin-resistant clones were isolated, and genomic sequences and protein expression of target genes were analyzed using DNA sequencing and immunoblot analysis, respectively.

### Immunoprecipitation and pull-down assay

Immunoprecipitation and pull-down assay were performed as described previously (19). For immunoprecipitation, cell lysates were precleared with protein G Sepharose 4 Fast Flow (Cytiva) for 30 min at 4 °C. Precleared lysates were then incubated with 4 μg of antibody bound to protein G Sepharose 4 Fast Flow for 2 h at 4 °C. For immunoprecipitation of ubiquitinated proteins, anti-FLAG antibody was used as control antibody (Fig. 5). For pull-down assay, GST fusion protein was produced in *E. coli* harboring pGEX-4T-3-3BP2-SH2-WT or -R486 K and purified using glutathione-Sepharose 4B (GSH beads). After cell lysates were precleared with GSH beads for 30 min at 4 °C, 20 μg of GST fusion protein bound to GSH beads were incubated with precleared lysates for 2 h at 4 °C. Immunoprecipitates or the GSH beads were then washed four times with the lysis buffer. After addition of SDS sampling buffer, immunoprecipitates or proteins bound to the GSH beads were incubated at 95 °C for 5 min and subjected to immunoblotting analysis.

### Lentivirus infection

Lentivirus infection was performed as previously described (28). Briefly, HEK-293T cells were transiently transfected with pLenti-CMV-GFP-Puro (658-5), pLenti-CMV-HA-3BP2-Puro, pLenti-CMV-HA-3BP2 R342A-Puro, pLenti-CMV-HA-3BP2 R262A/R342A-Puro, pLKO.1-puro shRNA-lacZ, pLKO.1-puro shRNA-HS1 #1, or pLKO.1-puro shRNA-HS1 #2, together with psPAX2 and pCMV-VSV-G to generate lentiviruses. At 48 h after transfection, the cell culture supernatant was collected and filtered through a 0.45 μm pore size syringe filter followed by the addition of PEG to concentrate virus particles. Freshly isolated bone marrow cells (day 0) were infected with lentivirus on day 2, and the cell culture medium was replaced with growth medium containing 7.5 μg/ml puromycin on day 4 to select lentivirus-infected cells. On day 6 or 7 (for knockdown experiments), the cells were harvested and were subjected to experiments.

### Purification of total RNA, generation of first-strand cDNA, and qPCR

Total RNA (0.5 μg) purified using acid guanidinium thiocyanate-phenol-chloroform extraction was used to generate first-strand cDNA using the ReverTra Ace qPCR RT Master Mix with gDNA Remover (Toyobo) according to the manufacturer’s instructions. qPCR was performed using the KAPA SYBR Fast qPCR Kit (Kapa Biosystems) and analyzed using the QuantStudio 5 Real-Time PCR System (Thermo Fisher Scientific). The sequences of primer sets were as follows: *Ereg* (forward: 5′-GGCAGTTATCAGCACAACCG-3′, reverse: 5′-CATCGCAGACCAGTGTAGCC-3′), *Tnfsf15* (forward: 5′-ATGCTTCGGGCCATAACAGA-3′, reverse: 5′-TGAAGGCCATCCCTAGGTCA-3′), *Tnf* (forward: 5′-CACGTCGTAGCAAACCACCAAGTGGA-3′, reverse: 5′-TGGGAGTAGACAAGGTACAACCC-3′), *Il1b* (forward: 5′-CAACCAACAAGTGATATTCTCCATG-3′, reverse: 5′-GATCCACACTCTCCAGCTGCA-3′), *Il10* (forward: 5′-TGAGGCGCTGTCGTCATCGATTTCTCCC-3′, reverse: 5′- ACCTGCTCCACTGCCTTGCT-3′), and *Gapdh* (forward: 5′- TTCACCACCATGGAGAAGGC-3′, reverse: 5′- GGCATGGACTGTGGTCATGA-3′).

### Identification of proteins interacting with the SH2 domain of 3BP2 by DIA-MS using nanoflow liquid chromatography-MS/MS

DIA-MS was carried out by Kazusa Genome Technologies as described previously (66). In brief, after proteins bound to GSH beads were digested with 5 μg/ml trypsin (Trypsin Platinum, Promega) for 14 h at 37 °C, the obtained peptides were injected into a 75 μm × 12 cm nanoflow liquid chromatography column (Nikkyo Technos) and separated using a 70-min gradient (A; 0.1% formic acid in water, B; 0.1% formic acid in 80% acetonitrile) of 6% B at 0 min, 32% B at 62 min, 75% B at 68 min, and 75% B at 70 min at a flow rate of 0.2 μl/min using an UltiMate 3000RSLCnano LC system (Thermo Fisher Scientific). The peptides eluted from the column were further analyzed with a Q Exactive HF-X system (Thermo Fisher Scientific). MS1 spectra were collected in the 495 to 745 m/z range at a resolution of 30,000 to set an automatic gain control target of 3 × 10^6^ and maximum injection time of 55 ms. MS2 spectra were collected at more than 200 m/z at a resolution of 30,000 to set an automatic gain control target of 3 × 10^6^, maximum injection time set at “Auto,” normalized collision energy of 23%, and isolation window of 4.0 thomson. For the identification and quantification of proteins, the MS data were analyzed using the DIA-NN software (version 1.81, https://github.com/vdemichev/DiaNN) employing a predicted spectral library generated from the mouse protein sequence in the UniProt database (Proteomes ID: UP000000589, https://www.uniprot.org/). The parameters for generating the spectral library were as follows: “FASTA digest for library-free search/library generation” was enabled; “deep learning-based spectra, RTs, and IMs prediction” was enabled; the digested enzyme was trypsin, with a missed cleavage allowance of 1; “N-term M excision” was enabled; “C-terminal carbamidomethylation” was enabled; the peptide length range was from 7 to 45; the precursor charge range was between 2 to 4; the precursor m/z range was from 490 to 750; and the fragment ion m/z range was from 200 to 1800. The DIA-NN search parameters have been described previously (66).

### Statistical analyses

Statistical significance was determined using an unpaired two-tailed Student’s *t* test or unpaired two-tailed Welch’s *t* test for two groups of data. One-way or two-way ANOVA, followed by Dunnett’s multiple comparison test or the Tukey–Kramer test, was used for more than two groups of data. All statistical analyses were performed using the R software version 4.32 (https://www.r-project.org/).

## Data availability

All data obtained in this study are included in the main figures and supporting information. Data obtained from the microarray analysis were deposited in the Gene Expression Omnibus repository (GSE263167). The raw data of MS and files generated by DIA-NN are available at the jPOST repository (#JPST003225, https://repository.jpostdb.org/).

## Supporting information

This article contains supporting information.

## Conflicts of interest

The authors declare that they have no conflicts of interest with the content of this article.
