# Supplementary material for: Adaptor protein 3BP2 regulates gene expression in addition to the ubiquitination and proteolytic activity of MALT1 in dectin-1–stimulated cells
Source: J Biol Chem. 2024 Nov 13;300(12):107980. doi: 10.1016/j.jbc.2024.107980 (PMC11647625; doi:10.1016/j.jbc.2024.107980)
Supplement: Supporting information [file mmc4.pdf]

Supporting Information

Adaptor protein 3BP2 regulates gene expression in addition to the ubiquitination and proteolytic activity of MALT1 in dectin-1-stimulated cells

Ayumi Tsubokawa, Kazuyasu Chihara, Yuri Chihara, Kenji Takeuchi, Shigeharu Fujieda, and Kiyonao Sada

Supporting information includes Table S1, Table S2, Table S3, Figure S1, Figure S2, Figure S3, and Figure S4.

Table S1 (a separate file)

A complete list of dectin-1-regulated genes (2,883 genes) was identified using the microarray analysis (Figure 1). The transcript cluster ID, average of expression level (Avg), standard deviation, fold change, *P*-value, false discovery rate (FDR) *P*-value, gene symbol, gene description, and group (type of transcript cluster) corresponding to an individual gene expressed in BMDCs obtained from wild-type mice without (Cd– WT cells) or with (Cd+ WT cells) stimulation with curdlan are summarized in a single Excel file.

Table S2 (a separate file)

A complete list of 3BP2 target genes (347 genes) was identified using the microarray analysis (Figure 1). The transcript cluster ID, average of expression level (Avg), standard deviation, fold change, *P*-value, false discovery rate (FDR) *P*-value, gene symbol, gene description, and group (type of transcript cluster) corresponding to an individual gene expressed in curdlan-stimulated BMDCs obtained from wild-type mice (Cd+ WT cells) or $3BP2^{DL/DL}$ mice (Cd+ DL cells) are summarized in a single Excel file.

Table S3 (a separate file)

A complete list of proteins co-precipitated with GST-3BP2-SH2 bound to GSH beads was identified using DIA-MS (Figure 8). Data were obtained from an individual biological replicate of a single experiment. The identification number, number of grouped proteins, accession number, master accession number, master protein name, master gene symbol, species, molecular weight (kDa), number of peptides, number of unique peptides, relative amount of protein precipitated with GST-3BP2-SH2-WT (3BP2-SH2-WT) or GST-3BP2-SH2-RK (3BP2-SH2-RK), and fold change (3BP2-SH2-WT/3BP2-SH2-RK, NA=not applicable) are summarized in a single Excel file.

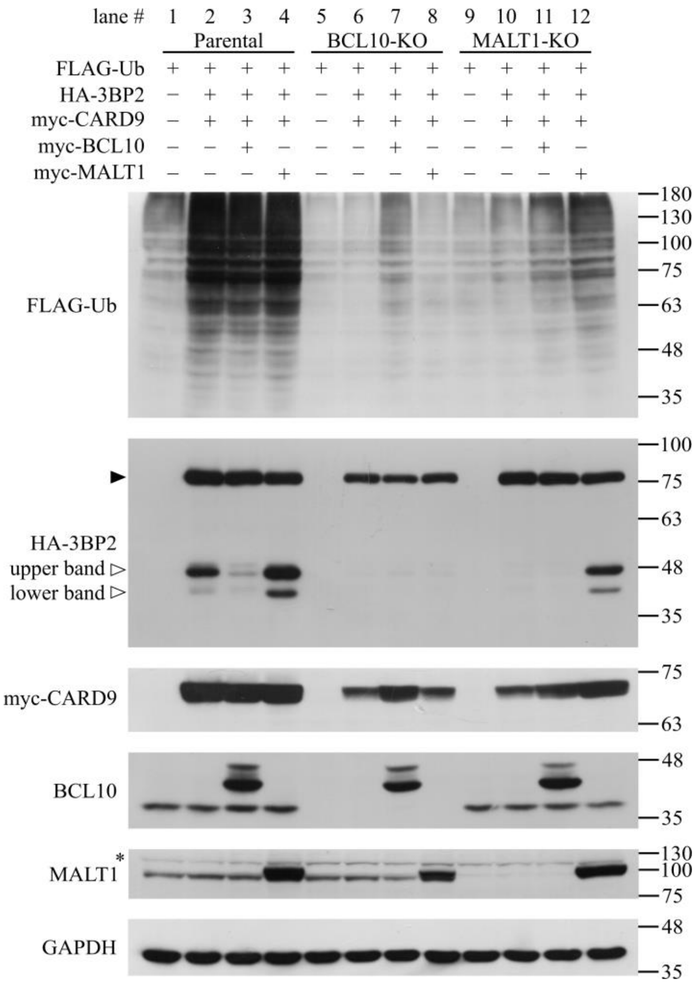

Figure S1. BCL10 and MALT1, in addition to CARD9, are required for 3BP2-mediated ubiquitination of cellular proteins in HEK-293T cells.

HEK-293T cells (Parental) or knockout (KO) cell lines were transfected without (−) or with (+) plasmids encoding the indicated proteins, and the cell lysates were subjected to immunoblotting analysis. The positions of the uncleaved or cleaved HA-3BP2 are indicated by closed or open arrowheads (upper and lower bands), respectively. * indicates the position of a protein nonspecifically recognized by the primary antibody. Data are representative of an individual biological replicate of three independent experiments.

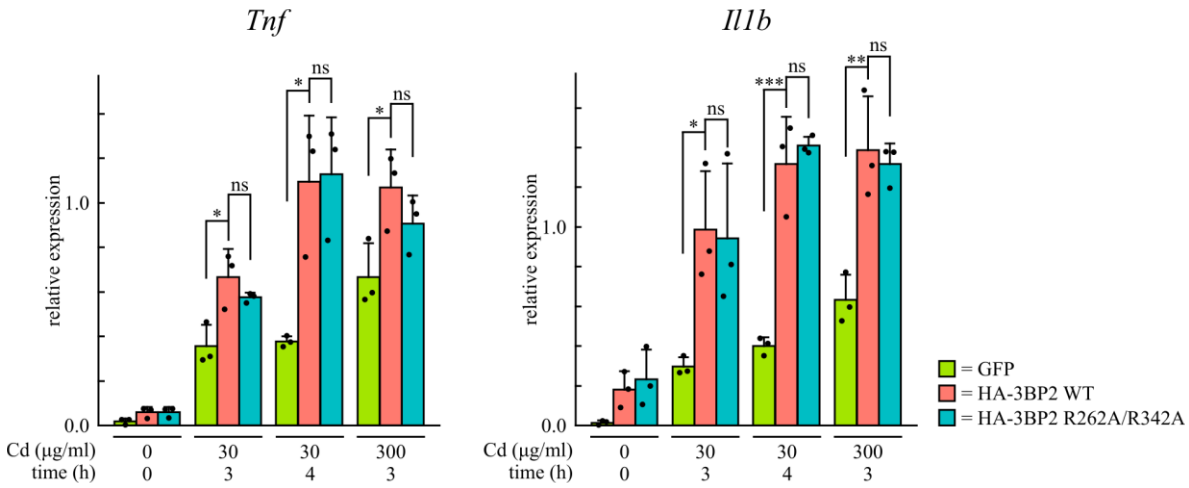

Figure S2. Expression of cytokine genes is not influenced by MALT1-dependent cleavage of 3BP2 in BMDCs differentially stimulated with curdlan.

BMDCs obtained from wild-type mice were infected with lentivirus for exogenous expression of the indicated proteins. After the cells were stimulated for 0, 3, or 4 h with cell culture plates coated with the indicated concentrations of curdlan, total RNA was extracted and the mRNA expression of cytokines was analyzed using qPCR. Closed circles correspond to an individual biological replicate of three independent experiments. Data are presented as the mean ± SD. Statistical significance was analyzed using one-way ANOVA followed by Dunnett's multiple comparison test. ***$P < 0.001$, **$P < 0.01$, or *$P < 0.05$ was considered statistically significant. ns = not significant.

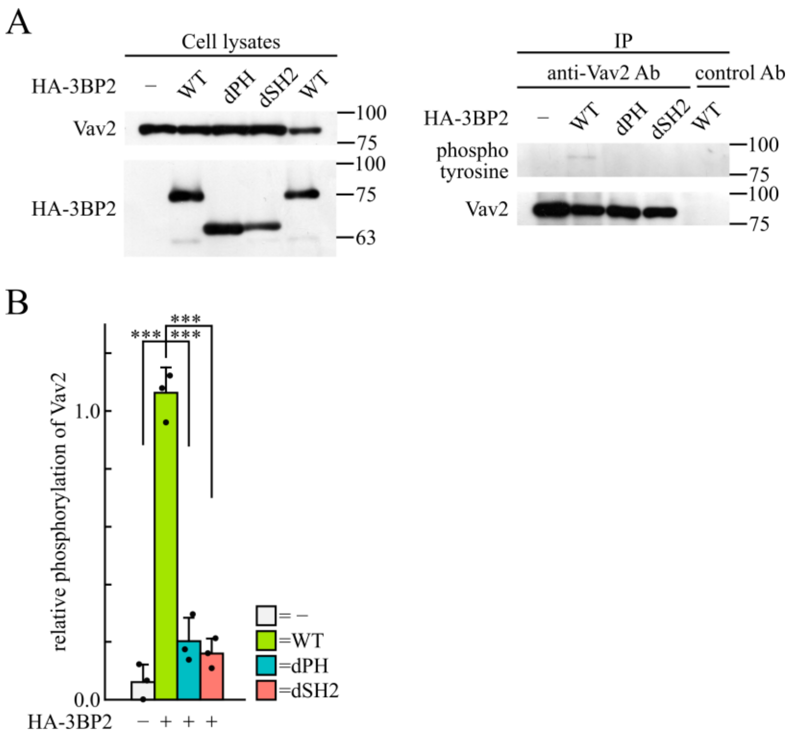

Figure S3. Overexpression of 3BP2 induces tyrosine phosphorylation of Vav2 endogenously expressed in HEK-293T cells.

(A) HEK-293T cells were transfected without (−) or with (+) plasmids encoding the indicated proteins, and endogenous Vav2 was immunoprecipitated from the cell lysates using the anti-Vav2 antibody (Ab) or control Ab. The cell lysates and immunoprecipitates were subjected to immunoblotting analysis. Data are representative of an individual biological replicate of three independent experiments. (B) Relative phosphorylation of Vav2 in immunoprecipitates was quantified using the immunoblots shown in panel A. Closed circles correspond to an individual biological replicate of three independent experiments. Data are presented as the mean ± SD. Statistical significance was analyzed using one-way ANOVA followed by Dunnett's multiple comparison test. ***$P < 0.001$ was considered statistically significant.

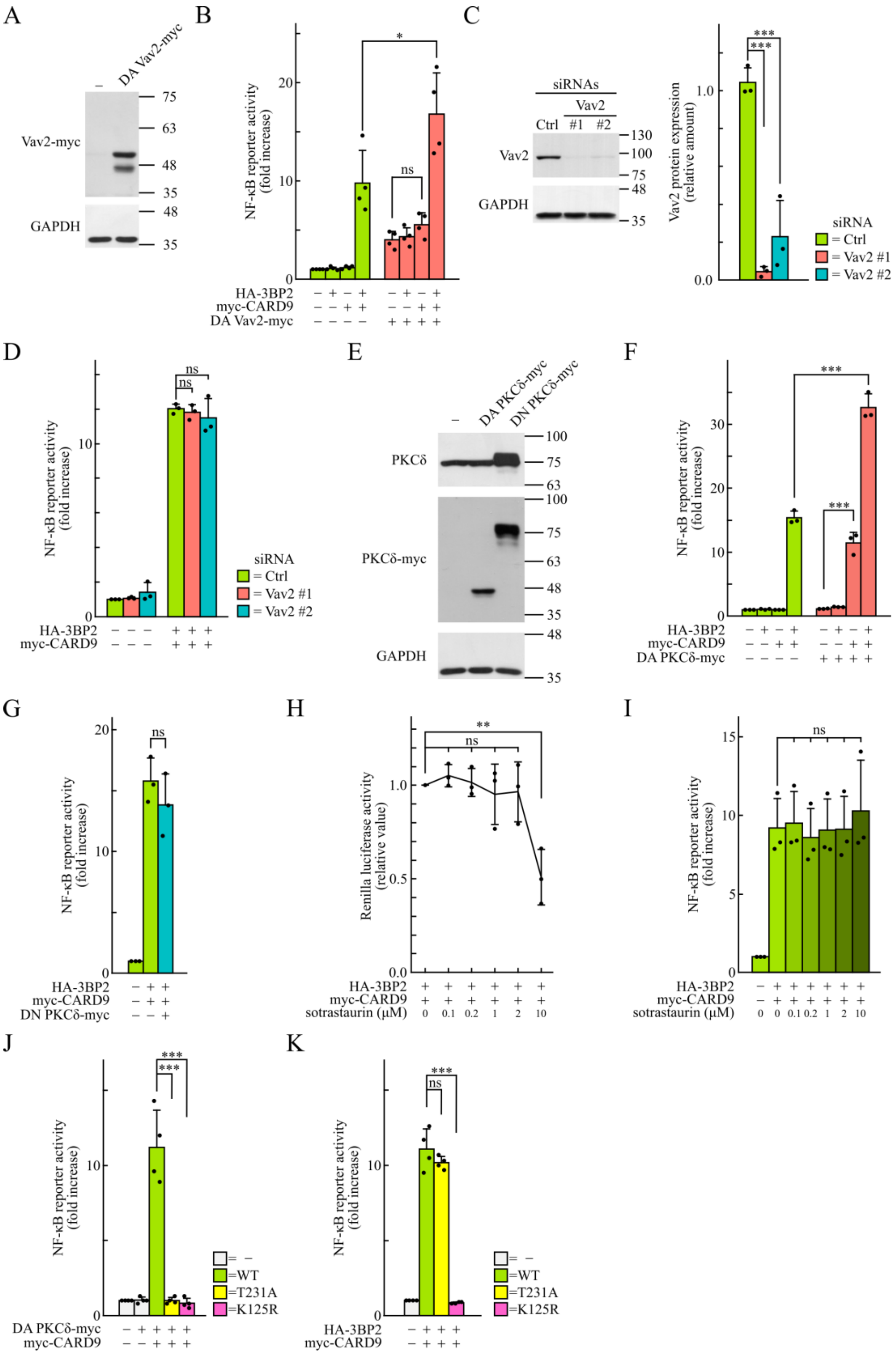
A
DA Vav2-myc
Vav2-myc
GAPDH
75
63
48
35
B
NF-κB reporter activity
(fold increase)
*
ns
HA-3BP2
myc-CARD9
DA Vav2-myc
C
siRNAs
Vav2
Ctrl
#1
#2
130
100
75
48
35
GAPDH
Vav2 protein expression
(relative amount)
***
siRNA
= Ctrl
= Vav2 #1
= Vav2 #2
D
NF-κB reporter activity
(fold increase)
ns
siRNA
= Ctrl
= Vav2 #1
= Vav2 #2
HA-3BP2
myc-CARD9
E
DA PKCδ-myc
DN PKCδ-myc
PKCδ
PKCδ-myc
GAPDH
F
NF-κB reporter activity
(fold increase)
***
HA-3BP2
myc-CARD9
DA PKCδ-myc
G
NF-κB reporter activity
(fold increase)
ns
HA-3BP2
myc-CARD9
DN PKCδ-myc
H
Renilla luciferase activity
(relative value)
**
ns
HA-3BP2
myc-CARD9
sotrastaurin (μM)
I
NF-κB reporter activity
(fold increase)
ns
HA-3BP2
myc-CARD9
sotrastaurin (μM)
J
NF-κB reporter activity
(fold increase)
***
=WT
=T231A
=K125R
DA PKCδ-myc
myc-CARD9
K
NF-κB reporter activity
(fold increase)
***
ns
=WT
=T231A
=K125R
HA-3BP2
myc-CARD9

Figure S4. Vav2 and PKCδ enhance the 3BP2-mediated activation of NF-κB in HEK-293T cells.

(A and E) HEK-293T cells were transfected without (−) or with (+) plasmids encoding the indicated proteins, and the cell lysates were subjected to immunoblotting analysis. Data are representative of an individual biological replicate of three independent experiments. DA Vav2-myc and DA PKCδ-myc represent the dominant active mutants of Vav2 and PKCδ, respectively, whereas DN PKCδ-myc indicates a dominant negative mutant of PKCδ. (B, D, F-K) HEK-293T cells were transfected with luciferase reporter plasmids without (−) or with (+) plasmids encoding the indicated proteins, and the cell lysates were subjected to a luciferase assay. (B, D, F, G, I-K) Normalized luciferase activities are expressed as fold increases relative to those of cells expressing GFP. Closed circles correspond to an individual biological replicate of three to six independent experiments. Data are presented as the mean ± SD. (C) HEK-293T cells were transfected with control (Ctrl), Vav2 #1, or Vav2 #2 siRNA. After 68 h, the relative expression of Vav2 in the transfected cells was analyzed using immunoblotting analysis. Left panels: representative immunoblots of an individual biological replicate of three independent experiments. Right panel: relative amounts of Vav2 (relative to GAPDH) quantified using the immunoblots shown in the left panels. Closed circles correspond to an individual biological replicate of three independent experiments. Data are presented as the mean ± SD. (H and I) After transfection, HEK-293T cells were cultured in the presence of indicated concentrations of a selective pan-PKC inhibitor, sotrastaurin, followed by luciferase assay. (H) Relative activities of Renilla luciferase in cells cultured with the indicated concentrations of sotrastaurin were determined. Closed circles correspond to an individual biological replicate of three independent experiments. Data are presented as the mean ± SD. Statistical significance was analyzed using two-way ANOVA followed by the Tukey–Kramer test (B and F), one-way ANOVA followed by Dunnett's multiple comparison test (C, D, H-K), or Student's *t*-test (G). ***$P < 0.001$, **$P < 0.01$, or *$P < 0.05$ was considered statistically significant. ns = not significant.
